# Lung‐Targeted Lipid Nanoparticle‐Delivered siUSP33 Attenuates SARS‐CoV‐2 Replication and Virulence by Promoting Envelope Degradation

**DOI:** 10.1002/advs.202406211

**Published:** 2024-09-20

**Authors:** Yuzheng Zhou, Yujie Liao, Lujie Fan, Xiafei Wei, Qiang Huang, Chuwei Yang, Wei Feng, Yezi Wu, Xiang Gao, Xiaotong Shen, Jian Zhou, Zanxian Xia, Zheng Zhang

**Affiliations:** ^1^ Institute for Hepatology National Clinical Research Center for Infectious Disease Shenzhen Third People's Hospital The Second Affiliated Hospital School of Medicine Southern University of Science and Technology Shenzhen 518112 China; ^2^ Department of Cell Biology School of Life Sciences Central South University Changsha 410083 China; ^3^ Guangzhou Laboratory Guangzhou 510700 China; ^4^ Hunan Key Laboratory of Animal Models for Human Diseases Hunan Key Laboratory of Medical Genetics & Center for Medical Genetics School of Life Sciences Central South University Changsha 410013 China; ^5^ Shenzhen Research Center for Communicable Disease Diagnosis and Treatment Chinese Academy of Medical Sciences Shenzhen 518112 China

**Keywords:** deubiquitinase, envelope (E), lipid nanoparticles, SARS‐CoV‐2, USP33

## Abstract

As a structural protein of SARS‐CoV‐2, the envelope (E) protein not only plays a key role in the formation of viral particles, but also forms ion channels and has pathogenic functions, including triggering cell death and inflammatory responses. The stability of E proteins is controlled by the host ubiquitin‐proteasome system. By screening human deubiquitinases, it is found that ubiquitin‐specific protease 33 (USP33) can enhance the stability of E proteins depending on its deubiquitinase activity, thereby promoting viral replication. In the absence of USP33, E proteins are rapidly degraded, leading to a reduced viral load and inflammation. Using lipid nanoparticle (LNP) encapsulation of siUSP33 by adjusting the lipid components (ionizable cationic lipids), siUSP33 is successfully delivered to mouse lung tissues, rapidly reducing USP33 expression in the lungs and maintaining knockdown for at least 14 days, effectively suppressing viral replication and virulence. This method of delivery allows efficient targeting of the lungs and a response to acute infections without long‐term USP33 deficiency. This research, based on the deubiquitination mechanism of USP33 on the E protein, demonstrates that LNP‐mediated siRNA delivery targeting USP33 plays a role in antiviral and anti‐inflammatory responses, offering a novel strategy for the prevention and treatment of SARS‐CoV‐2.

## Graphical abstract

The envelope (E) protein of SARS‐CoV‐2 hijacks the deubiquitinase ubiquitin‐specific protease 33 (USP33) in host cells to cleave off K48‐typed polyubiquitin chains and antagonize E3 ubiquitin ligase RNF5‐mediated degradation. Lipid nanoparticles target the delivery of siUSP33 to the lungs, effectively knock down USP33, and promote the proteasome‐dependent degradation of the E protein, inhibiting viral replication and attenuating virulence.

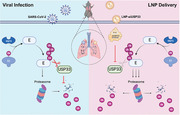

## 1 Introduction

The structure of SARS‐CoV‐2 comprises four structural proteins: spike (S), nucleocapsid (N), membrane (M), and envelope (E) protein.^[^ ^1^ ^]^ Among them, the S protein mainly binds to the receptor of the host cell and mediates the invasion of the virus,^[^ ^2^ ^]^ the N protein encapsulates and protects the viral RNA,^[^ ^3^, ^4^ ^]^ and the M protein forms the external enveloped structure of the viral particles, which participates in the assembly and release of the virus.^[^ ^5^, ^6^ ^]^ The E protein, the smallest one among the structural proteins containing only 75 amino acids, constitutes a very small proportion of viral particles, but is abundantly expressed in infected cells and is critical for viral virulence.^[^ ^7^ ^]^ E proteins are involved in virus assembly and the maintenance of virus particles by binding to M proteins.^[^ ^8^ ^]^ In addition, E proteins can form ion channels that affect the integrity of the cell M and the ionic balance inside and outside the host cell, causing cell death.^[^ ^9^ ^]^ Moreover, E proteins promote the formation and activation of NLRP3 inflammatory vesicles, leading to the release of inflammatory factors and the exacerbation of inflammatory response.^[^ ^10^, ^11^, ^12^ ^]^

The ubiquitin‐proteasome system is an important intracellular protein degradation pathway that rapidly recognizes and degrades certain viral proteins to mitigate the pathogenic effects of viruses.^[^ ^13^, ^14^ ^]^ ORF6, ORF9b, NSP5, and NSP8 of SARS‐CoV‐2,^[^ ^15^, ^16^, ^17^, ^18^ ^]^ and ORF3 as well as ORF4b of MERS‐CoV^[^ ^19^, ^20^ ^]^ can all be labeled with polyubiquitin chains and degraded by the proteasome in a short time. Ubiquitination involves an enzymatic cascade mediated by the E1‐activating enzyme, E2‐conjugating enzyme, and a large class of ≈600 E3 ubiquitin ligases.^[^ ^21^, ^22^ ^]^ Conversely, a group of deubiquitinases (Dubs) quickly cleave ubiquitin to reverse this process, and viruses can hijack Dubs in the host to resist degradation.^[^ ^23^, ^24^ ^]^ Therefore, the discovery of Dubs capable of stabilizing viral proteins and intervening in this process is a promising antiviral strategy. Ubiquitin‐specific protease 33 (USP33) is a Dub that has been implicated in the modulation of oncogenic signaling pathways, making it a potential target for cancer therapy.^[^ ^25^, ^26^, ^27^, ^28^ ^]^ In addition to its significance in cancer development and progression, emerging evidence has highlighted its multiple functions in neurodegenerative diseases, inflammation, and metabolic disorders,^[^ ^29^, ^30^, ^31^, ^32^ ^]^ expanding the potential impact of targeting Dub in various pathological conditions. However, the role of USP33 in viral infection and replication remains largely unknown.

Since the approval of lipid nanoparticles (LNPs) by the FDA, they have emerged as the preferred drug delivery platform within the biopharmaceutical industry. LNPs have been effectively utilized for the delivery of mRNA/siRNA to specific organs for the treatment of inflammation,^[^ ^33^ ^]^ viral infections,^[^ ^34^, ^35^, ^36^ ^]^ and cancer.^[^ ^37^, ^38^ ^]^ The selective organ targeting (SORT) delivery platform implements specific delivery to lung tissue based on physicochemical tropism/targeting.^[^ ^39^, ^40^, ^41^ ^]^ By modifying the classes or ratios of the components used in LNPs, it is feasible to convert LNPs to a positively charged state, thereby enhancing their lung‐targeting capabilities.^[^ ^42^, ^43^ ^]^ 1,2‐dioleoyl‐3‐trimethylammonium‐propane (DOTAP)‐LNPs possess lung‐specific targeting capabilities because of their interaction with the endogenous ligand vitronectin.^[^ ^44^, ^45^, ^46^ ^]^ Despite their numerous potential advantages, recent studies have revealed a previously unrecognized and significant adverse effect associated with DOTAP‐LNPs: their ability to induce thrombosis. However, by reducing the size of DOTAP‐LNPs to ≈100 nm, this side effect can be mitigated by preventing fibrinogen binding and clot formation.^[^ ^47^ ^]^ In our research, we established a potent platform for tailored SARS‐CoV‐2 infection control via SORT LNP‐based lung‐targeted (by adding the supplementary cationic lipid DOTAP at specific molar ratios (0–50%)) delivery of CY5‐modified siRNA, which could be a promising strategy for SARS‐CoV‐2 treatment.

In this study, we screened a cDNA library of human Dubs and identified USP33 as a host factor that promotes viral replication. Further investigation revealed that USP33 interacts with E proteins, removing K48‐typed polyubiquitin chains and enhancing E protein stability, thereby increasing its ability to induce cell death and inflammatory responses. In the absence of a specific USP33 inhibitor, DOTAP‐containing LNPs were used to encapsulate siUSP33. This delivery strategy efficiently targeted mouse lungs and specifically induced the knockdown of the USP33 protein, resulting in the effective inhibition of viral replication and inflammatory responses. Taken together, our study revealed a novel mechanism by which SARS‐CoV‐2 hijacks Dub USP33 to enhance viral replication and virulence, a promising target that could potentially be exploited to develop new antiviral therapies.

## 2 Results

### 2.1 Dubs Screening Identified USP33 as an Important Factor in Promoting Viral Replication by Targeting E

**Figure 1:**
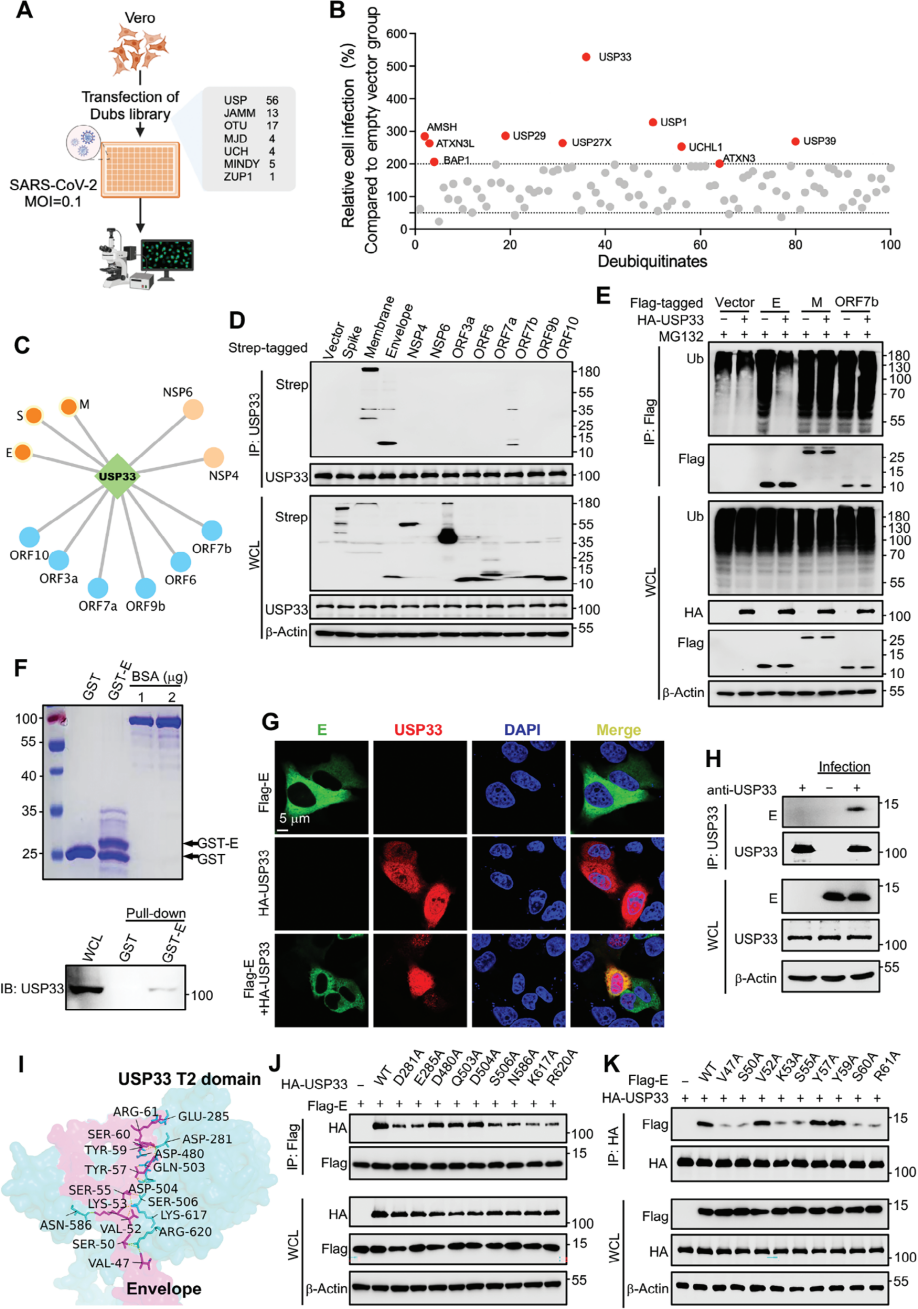
Dubs screening identified USP33 as an important factor in promoting viral replication by targeting E. A) Dubs screen workflow. The human Dubs overexpression library was transfected into Vero cells, followed by infection with SARS‐CoV‐2 at MOI = 0.1. 24 h later, the relative virus abundances were quantified via immunofluorescence detection of N protein. B) Results of the Dubs screens revealed USP33 as the top hit for positively regulating the replication of SARS‐CoV‐2. C) Protein interaction maps based on published databases showing viral proteins that potentially bind to USP33. D) HEK293T cells were transfected with indicated plasmids, and the whole cell lysates (WCL) were immunoprecipitated by anti‐USP33 antibody. The WCLs and precipitated samples were used for immunoblotting to detect the indicated proteins. E) HEK293T cells were co‐transfected with HA‐USP33 and Flag‐tagged viral genes, and treated with MG132 (10 µM). The WCLs were denatured and immunoprecipitated by anti‐Flag beads, followed by immunoblotting to detect indicated proteins. F) The GST and GST‐E proteins were expressed in BL21 and precipitated by anti‐GST beads. The beads were then incubated with cell lysates of HEK293T cells, followed by immunoblotting analysis. G) HeLa‐ACE2 cells were transfected with plasmids expressing Flag‐E and HA‐USP33, and immunofluorescence was performed with the indicated antibodies. Scale bars, 5 µm. H) HeLa‐ACE2 cells infected with SARS‐CoV‐2 at a MOI of 0.1 or not. 48 h later, the cells were lyzed and immunoprecipitated by anti‐USP33. The indicated proteins were detected via immunoblotting. I) The predicted interaction sites of USP33 and SARS‐CoV‐2 E proteins by AlphaFold 3. J,K) HEK293T cells were co‐transfected with indicated plasmids, followed by immunoprecipitation with anti‐Flag (J) or anti‐HA (K) beads. The WCLs and precipitated proteins were analyzed by immunoblotting.

SARS‐CoV‐2 utilizes the NSP3 protein with Dub activity for immune escape.^[^ ^48^ ^]^ To further select host Dubs for viral hijacking, we screened Vero cells using an overexpression library containing 100 Dubs via immunofluorescence (**Figure** **1**A). In addition to a number of Dubs that have been reported to regulate viral replication,^[^ ^49^, ^50^, ^51^ ^]^ we found that USP33 strongly promotes viral replication (Figure 1B). As Vero cells are naturally immunodeficient, we hypothesized that USP33 targets viral proteins to function as pro‐replication factors. By screening previous proteomic databases,^[^ ^52^ ^]^ we summarized the viral proteins that may interact with USP33, including structural proteins S, M, and E, nonstructural proteins NSP4 and NSP6, and accessory proteins ORF3a, ORF6, ORF7a, ORF7b, ORF9b, and ORF10 (Figure 1C). As verified by immunoprecipitation, M, E, and ORF7b significantly interacted with USP33, with the strongest binding occurring in E proteins (Figure 1D). USP33 acts as a Dub that removes the ubiquitin chains bound to substrate proteins. Therefore, by further in vivo ubiquitination assays, we found that although the E, M, and ORF7b proteins were significantly ubiquitinated, USP33 could only remove the polyubiquitin chains from the E protein (Figure 1E). When treated with cycloheximide (CHX), the E protein exhibited a half life of ≈16 h (Figure S1A, Supporting Information) and its degradation occurred exclusively via the proteasome (Figure S1B, Supporting Information). Although all structural proteins of SARS‐CoV‐2 had ubiquitination, only the E protein was relatively unstable and could be degraded within 12 h when treated with CHX (Figure S1C,D, Supporting Information).

The CO‐IP assay confirmed the existence of an interaction between USP33 and the E protein (Figure S1E–G, Supporting Information). The interaction between E and USP33 was further confirmed by purifying E protein from *E. coli* and performing GST pull‐down experiments (Figure 1F). Immunofluorescence experiments also revealed the colocalization of E and USP33 in cells (Figure 1G). Indeed, USP33 was immunoprecipitated together with the E protein in SARS‐CoV‐2‐infected HeLa‐ACE2 cells (Figure 1H). USP33 is located in both the cytoplasm and the nucleus. In the cytoplasm, it participates mainly in the regulation of deubiquitination while in the nucleus it regulates transcription and DNA repair. Only cytoplasmic USP33 can interact with E proteins (Figure S2A, Supporting Information). Interestingly, when the HeLa‐ACE2 cells were infected with SARS‐CoV‐2, USP33 migrated from the nucleus to the cytoplasm, resulting in an increase in the amount of USP33 in the cytoplasm and a decrease in the nucleus (Figure S2B,C, Supporting Information). This is likely related to several other proteins that interact with USP33 such as M and ORF7b, resulting in USP33 being effectively utilized by E protein to antagonize its ubiquitinated degradation (Figure S2D, Supporting Information). Another reported E protein deubiquitinase, USP39, was enhanced in expression after viral infection.^[^ ^53^ ^]^ But in different cells such as A549‐ACE2 and Calu3 cells, we confirmed that total protein levels of USP33 were not changed in response to viral infection, suggesting a different strategy for E hijacking of USP33 from USP39 (Figure S2E, Supporting Information). Although the protein level of USP33 is not affected by viral infection, we compared the transcriptomics in severe patients, mild patients, and healthy populations, and found higher levels of USP33 in patients with COVID‐19, which reflected the impact of USP33 on disease severity (Figure S2F, Supporting Information).

USP33 is a multi‐domain protein, and we constructed truncated domains of USP33 to verify their interactions with the E protein. The results showed that the T2 domain (185‐716 aa) was sufficient to interact with the E protein (Figure S3A,B, Supporting Information). Moreover, we predicted more precise regions and sites of the interaction between E protein and USP33 T2 domain by AlphaFold 3. The results showed that the nine amino residues on each of the E and USP33 proteins were critical for mediating the interaction (Figure 1I; Figure S3C,D, Supporting Information). By mutating all predicated amino acid sites on the E and USP33 proteins (E‐9A and USP33‐9A), and performing CO‐IP analysis, we confirmed that either E‐9A or USP33‐9A can no longer bind to the other protein (Figure S3E,F, Supporting Information). Next, we constructed different single‐site mutants and further analyzed the effect of the different sites on the interaction between E and USP33. The results demonstrated that the D281, E285, S506, N586, K617, and K620 on the USP33 protein, and the V47, S50, K53, S55, S60, and R61 sites on the E protein were important for the interaction (Figure 1J,K).

### 2.2 USP33 Enhanced E Stabilization by Removing K48‐Typed Polyubiquitin Chains

**Figure 2:**
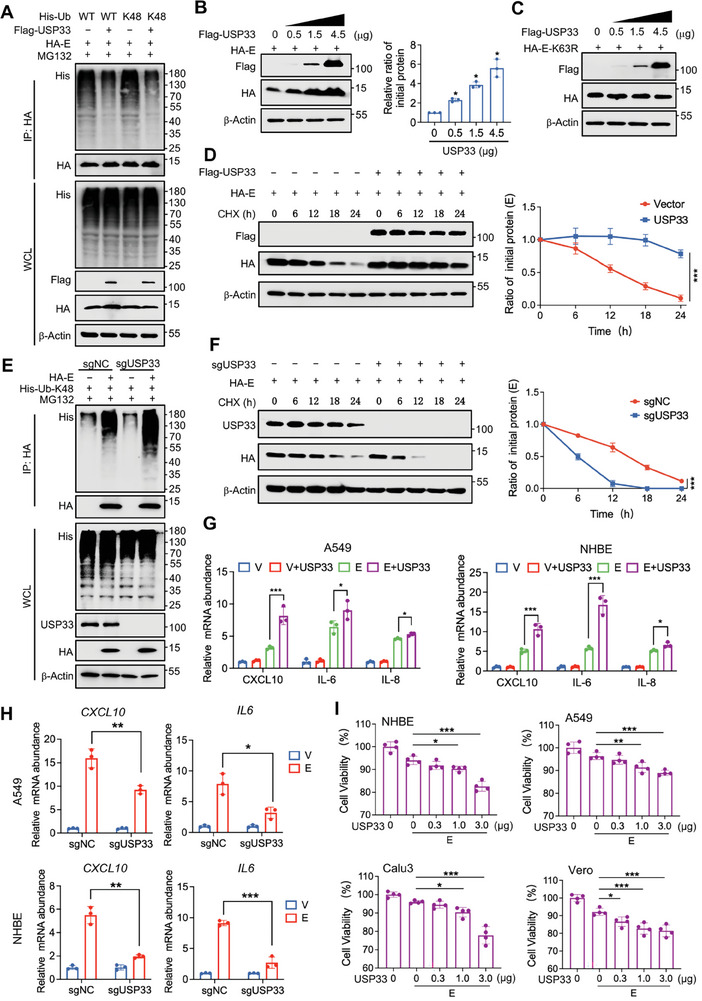
USP33 enhances E stabilization by removing K48‐typed polyubiquitin chains. A) HEK293T cells were transfected with indicated plasmids and treated with MG132. The WCLs were denatured and immunoprecipitated with anti‐HA beads, followed by immunoblotting. B) HEK293T cells expressing HA‐E were transfected with increasing amount of plasmids containing Flag‐USP33. The cells were lysed to detect the protein level of E by immunoblotting (left) and quantification relative to β‐Actin was shown (right). C) HEK293T cells expressing ubiquitination‐resistant mutant HA‐E‐K63R were transfected with increasing amount of plasmids containing Flag‐USP33. The protein levels of E‐K63R were analyzed via immunoblotting. D) HEK293T cells expressing HA‐E were transfected with empty vector or Flag‐USP33, treated with CHX, and collected at the indicated time for immunoblotting to analyze the protein level of E (left). Quantification relative to β‐Actin was shown (right). E) HEK293T cells transduced with lentivirus containing sgNC or sgUSP33 were then transfected with indicated plasmids and treated with MG132. The WCLs were denatured and immunoprecipitated with anti‐HA beads, followed by immunoblotting to analyze the polyubiquitinated chains of E protein. F) The protein level of E in HEK293T cells transfected with sgNC or sgUSP33 were analyzed at the indicated time with CHX treatment (left). Quantification relative to β‐Actin was shown (right). G) A549 and NHBE cells transfected with the indicated plasmids were lyzed to extract total RNA, and the indicated genes were analyzed using qRT‐PCR. H) A549 and NHBE WT and USP33‐KO cells were transfected with the indicated plasmids. Total RNA was extracted to analyze the relative mRNA level of *CXCL10* and *IL6*. I) NHBE, A549, Calu3, and Vero cells expressing E proteins or not were transfected with increasing amount of plasmids containing USP33. The cell viability was assayed using CCK‐8 reagent. Student's *t *test (unpaired, two‐tailed) was used to compare two independent groups, and a two‐way ANOVA test was performed for comparisons of multiple groups.  *p < 0.05; **p < 0.01; ***p < 0.001.

Proteins recognized and degraded by proteasomes generally form K48‐typed polyubiquitin chains. RNF5 is the major E3 ubiquitin ligase that catalyzes ubiquitination occurring at the K63 site of E protein.^[^ ^54^ ^]^ By screening different mutant types of ubiquitin, we identified the main type of ubiquitination on the E protein as the K48‐typed polyubiquitin chains, and USP33 significantly removed K48‐typed polyubiquitin chains from E proteins (**Figure** **2**A; Figure S4A, Supporting Information). When the K63 site of the E protein was mutated, the E protein was no longer ubiquitinated and was not subject to the deubiquitination regulation by USP33 (Figure S4B, Supporting Information). By screening previously identified interactomes, we discovered that E proteins had the ability to bind not only to USP33 but also to several other intracellular Dubs, including USP32 and USP39 (Figure S4C, Supporting Information).^[^ ^24^, ^52^ ^]^ USP39 also mildly enhanced the stability of the E protein, a phenomenon that aligns with the findings reported by Zhang et al.^[^ ^53^ ^]^ However, the effect of USP33 was more significant than that of USP39 (Figure 2B; Figure S4D, Supporting Information). In different lung‐associated cells, such as A549‐ACE2 and Calu3, overexpression of USP33 enhanced E protein levels more strongly than overexpression of USP39 (Figure S4E, Supporting Information). In addition, USP33 also had a stronger effect on promoting viral replication than USP39 (Figure S4F, Supporting Information). When a ubiquitination‐resistant mutation was introduced at K63, the influence of USP33 on E protein stability was no longer observed (Figure 2C). And USP33 overexpression notably extended the half‐life of E proteins (Figure 2D).

Furthermore, we constructed four shRNAs targeting USP33. Upon knockdown of USP33, the half life of the E protein was significantly shortened (Figure S5A,B, Supporting Information). Similarly, siRNA targeting USP33 reduced the stability of E proteins (Figure S5C,D, Supporting Information). Following USP33 knockdown using either shRNA or siRNA, the ubiquitination levels of E proteins increased (Figure S5E,F, Supporting Information). To further validate these findings, we engineered four sgRNAs and observed that in HEK293T cells with targeted USP33 knockout (KO), the ubiquitination level of E proteins was significantly increased and the half life was shortened (Figure 2E,F; Figure S5G, Supporting Information). To further determine the effect of USP33 on the pathological functions of E proteins, we analyzed the ability of E proteins to promote inflammation and induce cell death. In cell lines, such as A549 and NHBE, USP33 enhanced the ability of E proteins to induce the release of inflammatory factors (Figure 2G). We also confirmed that USP33 could enhance the ability of E proteins to induce inflammation more strongly than USP39 (Figure S5H, Supporting Information). In contrast, in USP33 KO cells, the ability of E proteins to induce the release of inflammatory factors was significantly weakened compared to that in WT cells (Figure 2H; Figure S5I, Supporting Information). Moreover, the Dub USP33 enhanced the ability of E proteins to induce cell death in a dose‐dependent manner, but had no effect on the ubiquitination‐resistant mutation K63R (Figure 2I; Figure S5J, Supporting Information). However, in A549 and NHBE cells in which RNF5 was knocked out, USP33 no longer had an effect on the ability of E proteins to induce cell death because E proteins were not ubiquitinated (Figure S5K,L, Supporting Information).

PLpro is the catalytic domain of the SARS‐CoV‐2 NSP3 protein with Dub activity that mediates the deubiquitination of multiple ubiquitinated proteins.^[^ ^55^, ^56^ ^]^ Immunoprecipitation analyses showed that E proteins interacted with PLpro as well as NSP3 proteins (Figure S6A,B, Supporting Information). Immunoprecipitation analysis further demonstrated that PLpro interacted with E protein in the cytoplasm (Figure S6C, Supporting Information). The presence of PLpro prolonged the half life of the E protein, whereas the regulation of E protein stability was lost when the enzyme active site was mutated (Figure S6D,E, Supporting Information). Similarly, the Dub activity was crucial for the ability of PLpro to remove ubiquitin chains from E proteins (Figure S6F, Supporting Information). E proteins can utilize viral PLpro and hijack the host Dub USP33 to remove their polyubiquitinated chains. When considering both Dubs, USP33 had a more obvious deubiquitination effect on E proteins than PLpro (Figure S6G, Supporting Information). Several PLpro inhibitors have been developed to effectively inhibit viral replication.^[^ ^57^ ^]^ When the cells were treated with a PLpro inhibitor, the enhancement of E protein stability and deubiquitination by PLpro was attenuated (Figure S6H–J, Supporting Information).

### 2.3 USP33 Can Facilitate SARS‐CoV‐2 Replication

**Figure 3:**
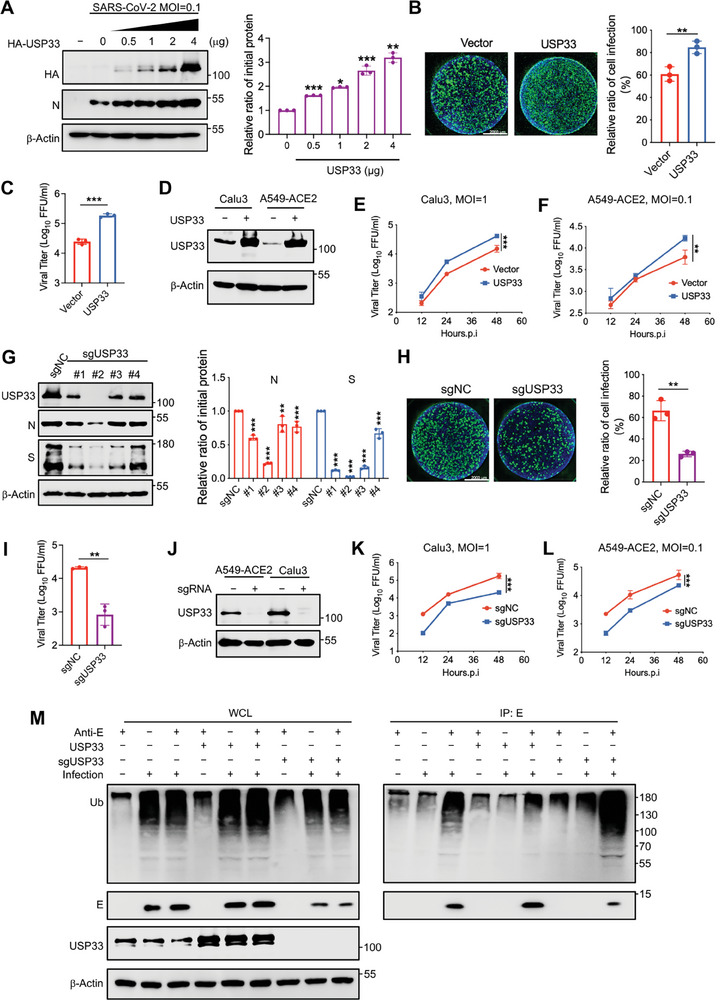
USP33 can facilitate SARS‐CoV‐2 replication. A) HeLa‐ACE2 cells transfected with increasing amount of plasmids containing HA‐USP33 were infected with SARS‐CoV‐2 at a MOI of 0.1. The protein levels of SARS‐CoV‐2 N were analyzed via immunoblotting (left) and quantified (right). B,C) HeLa‐ACE2 cells were transfected with vector or HA‐USP33 and infected with SARS‐CoV‐2 at a MOI of 0.1. The relative ratio of infection was analyzed via immunofluorescence (B, left) and quantification was shown as mean ± SD (n = 3 independent experiments) (B, right). Viral titers were measured using FFA (C). D) The USP33 was overexpressed in Calu3 and A549‐ACE2 cells via lentivirus transduction and protein levels were analyzed via immunoblotting. E,F) Calu3 (E) and A549 (F) cells overexpressing USP33 were infected with SARS‐CoV‐2 at the indicated MOIs. The viral kinetics were analyzed using FFA. G) HeLa‐ACE2 cells were transfected with sgNC and 4 sgRNAs targeting USP33, followed by infection with SARS‐CoV‐2 at a MOI of 0.1. The indicated protein levels were analyzed via immunoblotting (left) and quantification was shown (right). H,I) HeLa‐ACE2 cells transfected with sgNC or sgUSP33 were then infected with SARS‐CoV‐2 at a MOI of 0.1 for 24 h. The relative ratio of infection was analyzed via immunofluorescence (H, left) and quantification was shown as mean ± SD (n = 3 independent experiments) (H, right). Viral titers were measured using FFA (I). J) Calu3 and A549‐ACE2 cells were transduced with sgUSP33 by lentivirus and treated with puromycin (2 µg mL⁻^1^) for 48 h. The protein levels of USP33 were analyzed via immunoblotting. K,L) USP33 knockout Calu3 (K) and A549‐ACE2 (L) cells were infected with SARS‐CoV‐2 at indicated MOIs and the viral kinetics were analyzed using FFA. M) The A549‐ACE2 cells transduced with lentivirus containing empty vector, USP33, or sgUSP33 were then infected with SARS‐CoV‐2 at a MOI of 0.1 for 24 h. The WCLs were denatured and immunoprecipitated with anti‐SARS‐CoV‐2‐E antibody, followed by immunoblotting. Student's *t *test (unpaired, two‐tailed) was used to compare two independent groups, and a two‐way ANOVA test was performed for comparisons of multiple groups.  *P < 0.05; **P < 0.01; ***P < 0.001.

The stability of E proteins is critical for the replication of SARS‐CoV‐2.^[^ ^54^, ^58^ ^]^ To further verify the effect of USP33 on SARS‐CoV‐2 replication, we examined the effect of USP33 on HeLa‐ACE2 cells. With the gradient increase in the USP33 expression, there was a corresponding gradual increase in the amount of SARS‐CoV‐2 N protein, indicating augmentation of the viral load (**Figure** **3**A). Immunofluorescence and focus‐forming assay (FFA) further confirmed that USP33 overexpression enhanced viral replication capacity (Figure 3B,C). To confirm the facilitating effect of USP33 on viral replication under actual infection conditions, we selected two lung‐related cell lines, Calu3 and A549‐ACE2, and constructed corresponding cell lines stably overexpressing USP33 (Figure 3D). We then infected the cells with SARS‐CoV‐2 and assayed the progeny virus levels in the culture medium at 12, 24, and 48 h post infection. The results showed that after overexpressing USP33, compared to WT cells, the replication of SARS‐CoV‐2 in Calu3 and A549‐ACE2 cells was enhanced (Figure 3E,F).

Furthermore, we knocked down USP33 protein expression in HeLa‐ACE2 cells using shRNA (Figure S7A, Supporting Information). Immunofluorescence and FFA results showed that the viral replication capacity was weakened after USP33 knockdown (Figure S7B,C, Supporting Information). Additionally, we suppressed the expression of USP33 in A549‐ACE2 cells using siRNA targeting USP33. As the amount of siRNA transfected increased, the protein level of USP33 gradually decreased, and the immunofluorescence results showed that the viral load also decreased in a gradient manner (Figure S7D,F, Supporting Information). Viral kinetic analysis showed that when USP33 was knocked down, the replication ability of SARS‐CoV‐2 was weakened (Figure S7G, Supporting Information). To further clarify the role of USP33, we first knocked out USP33 in HeLa‐ACE2 cells using four sgRNAs and found that the protein level of USP33 was positively correlated with the viral load (Figure 3G). Immunofluorescence and FFA experiments showed that USP33 KO significantly inhibited viral replication in HeLa‐ACE2 cells (Figure 3H,I). Moreover, we constructed stable cell lines with USP33 KO in A549‐ACE2 and Calu3 cells (Figure 3J), and the viral load in USP33‐KO cells at different time points after infection was reduced compared to that in control cells (Figure 3K,L). The ubiquitination assay of the E protein in infected A549‐ACE2 cells showed that USP33 overexpression reduced the polyubiquitin chains of the E protein. In contrast, when USP33 was absent, the ubiquitination of E proteins was significantly increased (Figure 3M). These results strongly demonstrated the promoting effect of USP33 on viral replication via deubiquitination of the E protein.

### 2.4 USP33 Pro‐Replication Ability Depended on Its Dub Activity

**Figure 4:**
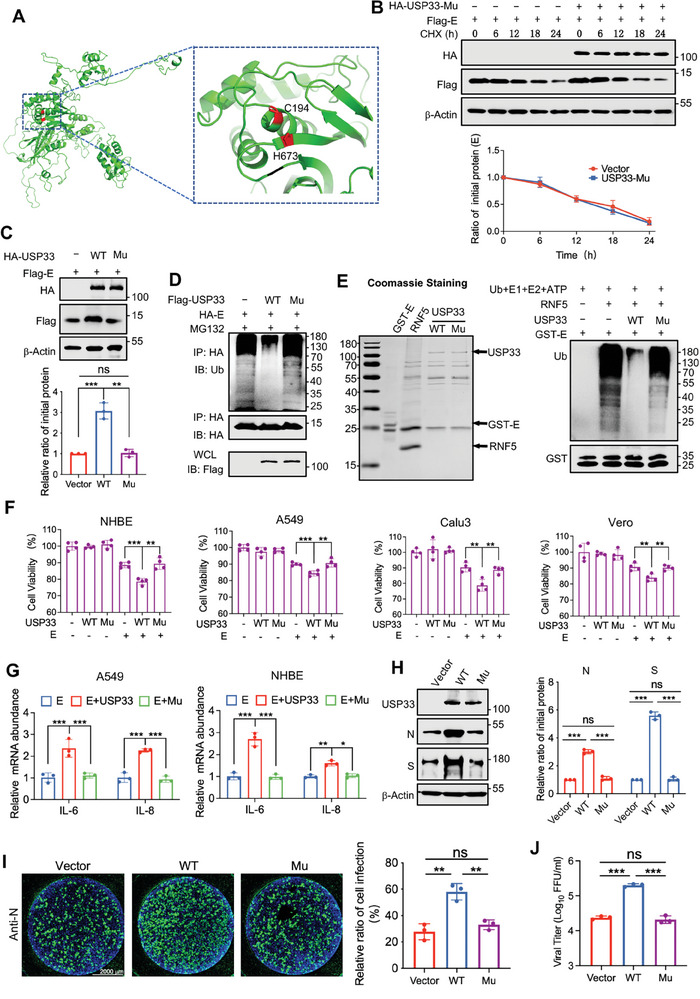
USP33 pro‐replication ability depended on its Dub activity. A) The structure of USP33 was modeled by the I‐TASSER server and visualized by PyMOL with two enzyme active sites highlighted in red. B) HEK293T cells expressing Flag‐E were transfected with empty vector or HA‐USP33‐Mu, treated with CHX, and collected at the indicated time for immunoblotting to analyze the protein level of E (up). Quantification relative to β‐Actin was shown (down). C) HEK293T cells expressing Flag‐E were transfected with empty vector, Flag‐USP33, or Flag‐USP33‐Mu and collected for analyzing the E protein level via immunoblotting (up). Quantification relative to β‐Actin was shown (down). D) HEK293T cells transfected with indicated plasmids were treated with MG132. The WCLs were denatured and immunoprecipitated with anti‐HA beads. The WCLs and precipitated proteins were analyzed via immunoblotting. E) The GST‐E protein purified from BL21, and Flag‐RNF5, Flag‐USP33 as well as Flag‐USP33‐Mu purified from HEK293T cells were quantified by Coomassie staining (left). The proteins were mixed in a reaction system with supplemented Ub, E1, E2, and ATP, and in vitro ubiquitination of E protein was detected via immunoblotting (right). F) NHBE, A549, Calu3, and Vero cells were transfected with indicated plasmids and cell viability was assayed with the CCK‐8 reagent. G) A549 and NHBE cells transfected with the indicated plasmids were lysed to extract total RNA, and the relative mRNA levels of *IL6* and *IL8* were analyzed using qRT‐PCR. H–J) HeLa‐ACE2 USP33‐KO cells were transfected with indicated plasmids and infected with SARS‐CoV‐2 at a MOI of 0.1 for 48 h. The protein levels (H), relative fluorescence intensity (I), and viral titers (J) were then analyzed. Quantification was shown as mean ± SD (n = 3 independent experiments). Student's *t *test (unpaired, two‐tailed) was used to compare two independent groups, and a two‐way ANOVA test was performed for comparisons of multiple groups.  *P < 0.05; **P < 0.01; ***P < 0.001.

The Dub activity of USP33 is dependent on two key sites in the UBP structural domain (C194 and H673) (**Figure** **4**A). To demonstrate that the role of USP33 in enhancing E protein stability and promoting viral replication relies on its Dub activity, we constructed an enzymatically inactive mutant (USP33‐Mu) by mutating two active sites (C194S/H673Q).^[^ ^59^ ^]^ USP33‐Mu retained the ability to bind to E proteins (Figure S8A, Supporting Information). However, unlike USP33‐WT, it no longer affected the half life and ubiquitination of the E protein (Figure 4B–D; Figure S8B, Supporting Information). Moreover, we purified the GST‐E protein from *E.coli*, and the E3 ubiquitin ligase RNF5, Dubs USP33‐WT as well as USP33‐Mu from HEK293T cells. In vitro ubiquitination assay showed that unlike USP33‐Mu, USP33‐WT could directly remove the K48‐typed polyubiquitin chains from the E protein (Figure 4E). To further demonstrate that SARS‐CoV‐2 can utilize Dub USP33 to enhance the pathogenic effect of E protein and virus replication, we first verified the ability of USP33 to enhance E protein‐promoted cell death in several cell lines, including NHBE, A549, Calu3, and Vero cells. The results showed that USP33 could not mediate cell death. However, in the presence of the E protein, unlike USP33‐Mu, USP33 overexpression significantly enhanced the proportion of cell death (Figure 4F). Furthermore, in A549 and NHBE cells, USP33 enhanced the ability of E protein to induce inflammatory responses, whereas USP33‐Mu did not (Figure 4G). In USP33‐KO HeLa‐ACE2 cells, the viral replication capacity significantly increased in cells replenished with USP33‐WT compared to those replenished with USP33‐Mu (Figure 4H–J).

E protein, an essential component of coronavirus particles, is found in all seven human coronaviruses. In addition to the high homology (89.3%) between SARS‐CoV and SARS‐CoV‐2, there were significant variations in the E proteins among other human coronaviruses (Figure S8C–E, Supporting Information). Although the half‐life analysis showed that all seven E proteins declined after 24 h when treated with CHX, the degradation of SARS‐CoV and SARS‐CoV‐2 E proteins was the most obvious. And USP33 only enhanced the stability of SARS‐CoV and SARS‐CoV‐2 E proteins (Figure S8F,G, Supporting Information). This is consistent with the fact that USP33 could only interact with the E proteins of SARS‐CoV and SARS‐CoV‐2, which was due to the interaction sites that we demonstrated were conserved in these two coronaviruses and existed as distinct amino acids in other coronaviruses (Figure S8H,I, Supporting Information).

### 2.5 LNP‐Encapsulated siUSP33 Can Be Targeted for Delivery to the Lungs

**Figure 5:**
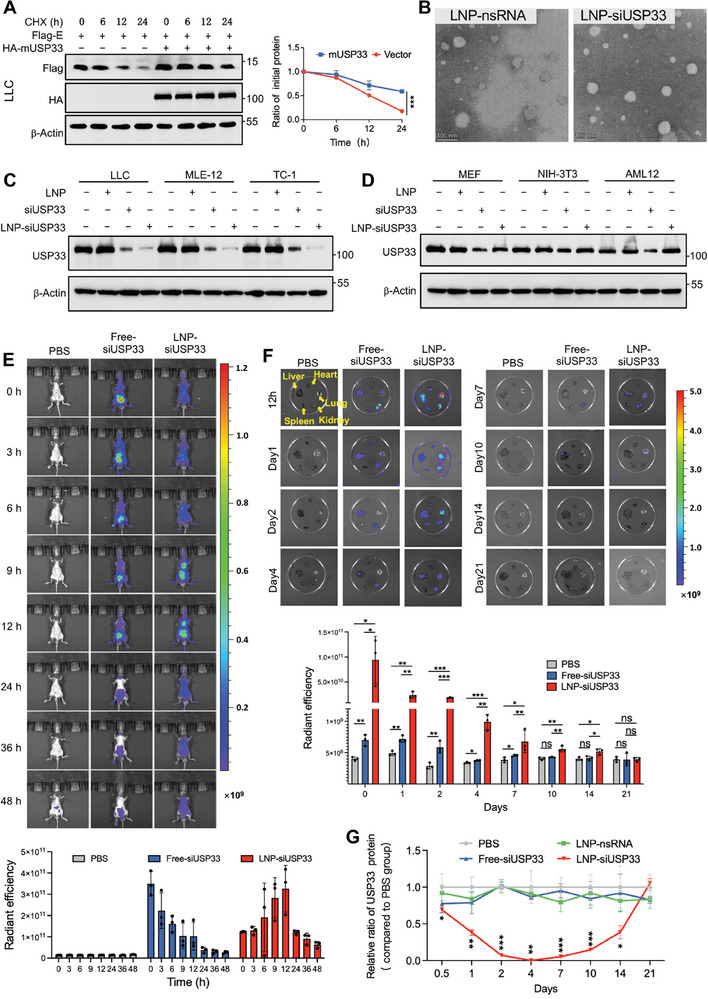
LNP‐encapsulated siUSP33 can be targeted for delivery to the lungs. A) LLC cells expressing Flag‐E were transfected with empty vector or HA‐mUSP33, treated with CHX and collected at the indicated time for immunoblotting to analyze the protein level of E. B) Representative transmission electron microscopic images of LNP‐nsRNA and LNP‐siUSP33. Scale bars, 100 nm. C,D) Lung‐associated cells (C) and non‐lung‐associated cells (D) were transfected with siRNA (100 pmol) or added LNP‐siRNA (0.132 µg) as indicated. Around 48 h later, the cells were collected for immunoblotting to detect the protein level of USP33. E) Representative whole‐body bioluminescence images of mice were measured via IVIS imaging system (up) and quantification was shown as mean ± SD (n = 3 independent experiments) (down). F) Representative ex vivo images of siRNA fluorescence in injected mouse organs were captured using the IVIS imaging system (up) and quantification was shown as mean ± SD (n = 3 independent experiments) (down). G) The lungs from mice of different treatments were collected at the indicated days, and the protein levels of USP33 in the lungs were quantified compared to the PBS‐treated group. Student's *t *test (unpaired, two‐tailed) was used to compare two independent groups, and a two‐way ANOVA test was performed for comparisons of multiple groups.  *P < 0.05; **P < 0.01; ***P < 0.001.

Given that the ability of USP33 to promote viral replication relies on its Dub activity, inhibition of the enzymatic activity of USP33 can effectively suppress viral replication. However, owing to high homology within the USP family, there are currently no specific inhibitors that target USP33.^[^ ^60^ ^]^ Therefore, we targeted the delivery of siUSP33 using LNP. With 89.5% homology between mouse and human USP33, we first demonstrated that mUSP33 could also enhance the stability of E proteins in mouse lung‐associated cells, including lung cancer cells (LLCs) and alveolar macrophages (MH‐S) (**Figure** **5**A; Figure S9A, Supporting Information). Four siRNAs targeting mouse USP33 were designed and screened for targeted knockdown with the highest efficiency (Figure S9B, Supporting Information). Typically, LNPs accumulate in the liver and kidneys, but LNPs with high molar ratios of ionizable cationic lipids show better targeting to the lungs. Therefore, we prepared siUSP33 encapsulated in DOTAP‐LNPs (LNP‐siUSP33), and characterized the size and surface charge of LNP‐encapsulated siRNA (Figure S9C, Supporting Information). The morphology of the LNP‐siRNA was further characterized via transmission electron microscopy, showing a well‐defined core structure (Figure 5B; Figure S9D, Supporting Information). And both LNP‐siUSP33 and LNP‐nsRNA are monodispersed spherical lipid nanoparticles with an apparent diameter of ≈110–140 nm and a morphological height of ≈10–15 nm, as can be seen in the 3D reconstructed version of the image constructed by atomic force microscopy (Figure S9E, Supporting Information). By transfecting free‐siUSP33 or adding LNP‐siUSP33 to mouse cells from different sources, we found that in lung‐related cells, including LLC, MLE‐12, and TC‐1, LNP‐siUSP33 had a higher knockdown efficiency than conventional siUSP33 transfection (Figure 5C). In non‐lung‐associated cells, such as MEF, NIH‐3T3, and AML12, the knockdown efficiency of LNP‐siUSP33 was reduced, whereas that of the transfection method was higher (Figure 5D).

We next compared several different ways of administering LNPs, including retro‐orbital injection, intravenous injection (Tail), and intranasal inoculation. The whole‐body bioluminescence imaging and ex vivo fluorescence detection showed the least loss and the most LNP‐siUSP33 delivered to the lungs of retro‐orbital injection (Figure S9F, Supporting Information). The retro‐orbital injection method also showed the best knockdown efficiency on day 1, day 2, and day 4 post‐treatment (Figure S9G, Supporting Information). To better analyze the effect of LNP‐siUSP33 delivery to the lungs and the efficiency of knocking down USP33 over time after injection, the mice were injected with PBS, Free‐siUSP33, and LNP‐siUSP33. Whole‐body bioluminescence imaging demonstrated that after injection, Free‐siUSP33 quickly spread throughout the body, gradually accumulating in the liver and kidneys, whereas LNP‐siUSP33, although also accumulated in small amounts in the liver and kidneys, gradually targeted the lungs (Figure 5E). On days 0.5, 1, 2, 4, 7, 10, 14, and 21 post‐injection, ex vivo fluorescence detection was performed on lungs, heart, liver, kidney, and spleen tissues. The results showed that LNP‐siUSP33 specifically targeted the lungs, lasted for ≈14 d, and was completely metabolized by day 21 (Figure 5F). Moreover, the protein level analysis showed that in the lungs, USP33 was significantly knocked down 1 day after injection, which persisted for at least 14 days, returning to the original levels by day 21 (Figure 5G; Figure S10A, Supporting Information). In the livers and kidneys, USP33 was only knocked down in the first 2 days and then returned to baseline levels, with no observed changes in USP33 protein levels in other organs (Figure S10B, Supporting Information).

Given that LNP delivery in vivo peaks on the first day post‐injection and USP33 knockdown efficiency is optimal four days post‐injection, we collected blood samples from each group on day 1 and day 4 for analysis of transcriptomics and regular physiological indexes. The results showed that there were no significant changes in the hematological indexes, and the liver and kidney functions of the mice were essentially normal (Figure S11, Supporting Information). In addition, peripheral blood transcriptomics results from different groups showed that although LNP injection (Figure S12, Supporting Information) or knockdown of USP33 (Figure S13, Supporting Information) resulted in changes in the transcription of some genes, these were not associated with the inflammatory response. This indicates that our strategy of utilizing Dlin‐MC3‐DMA to encapsulate siRNA and adding an additional cationic lipid (DOTAP), was effective in reducing side effects. This LNP system maintains a relatively neutral surface charge at physiological pH levels. We also kept its diameter below 140 nm to reduce the binding of LNP to fibrinogen and the formation of clots. The above results demonstrate that our LNP delivery strategy and knockdown of USP33 is a potential antiviral approach.

### 2.6 LNP‐siUSP33 Reduced Viral Load and Lung Pathology In Vivo

**Figure 6:**
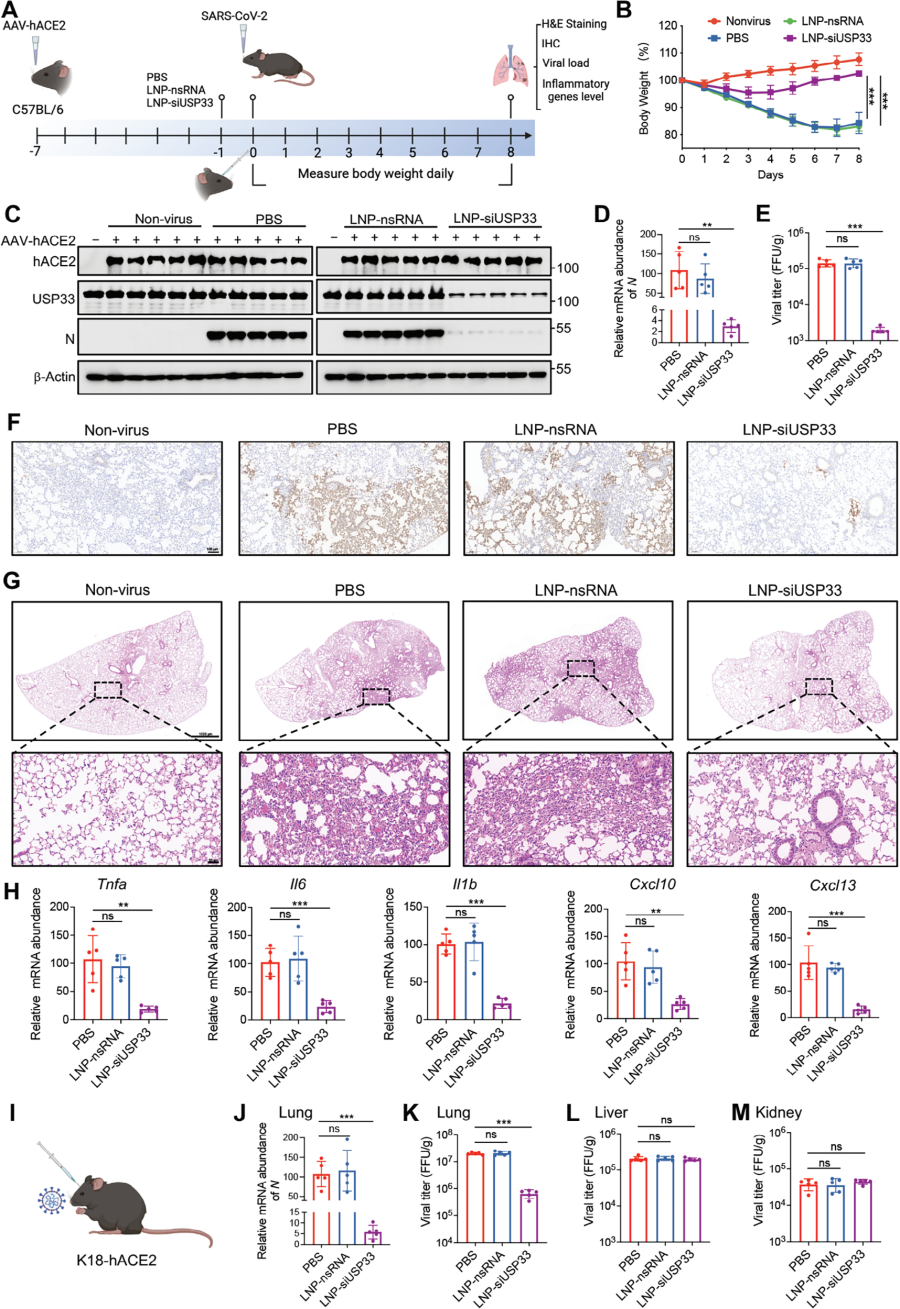
LNP‐siUSP33 reduces viral load and lung pathology in vivo. A) C57BL/6 mice were nasally infected with AAV‐hACE2 and injected with indicated reagents via retro‐orbital. On the second day of injection, the mice were infected nasally with 1 × 10^5^ FFU WT SARS‐CoV‐2. All the lung tissues were collected on the 8th day after infection. B) The body weights of the mice were recorded daily for 8 days after infection. Quantification of body weights compared to pre‐infection was shown as mean ± SD (n = 5 independent experiments). C) The lung tissues of mice were collected on the 8th day after infection and then lyzed to detect the levels of indicated proteins via immunoblotting. D) Total RNA was extracted from the lung tissues, and the relative levels of SARS‐CoV‐2 *N* genes were detected. E) The lungs were ground into homogenates and then infected Vero cells, and viral titers were calculated using the FFA. F) Immunohistochemistry analyses with anti‐SARS‐CoV‐2 N antibody was performed to assess the relative amount of SARS‐CoV‐2 in the lungs. Scale bar, 100 µm. G) H&E staining was performed to observe the intensity of the inflammatory infiltrate. Scale bars, 1000 µm (up) and 50 µm (down). H) Total RNA was extracted from the lungs and relative levels of indicated inflammatory genes were detected using qRT‐PCR. I) Schematic diagram of the K18‐hACE2 mice of SARS‐CoV‐2 infection. J) Total RNA was extracted from the lung tissues collected on day 4 after infection, and the relative levels of SARS‐CoV‐2 *N* genes were detected. K–M) The viral loads of the lungs (K), livers (L), and kidneys (M) collected on day 4 were calculated using FFA. Student's *t *test (unpaired, two‐tailed) was used to compare two independent groups, and a two‐way ANOVA test was performed for comparisons of multiple groups.  *P < 0.05; **P < 0.01; ***P < 0.001; ns, not significant.

To validate the antiviral effect of USP33 in mice in vivo, we overexpressed human ACE2 protein in the lungs of mice using AAV. Six days after AAV transduction, we injected PBS, LNP‐nsRNA, and LNP‐siUSP33 into mice via the retro‐orbital route. And the next day, we infected the mice with the wild‐type strain of SARS‐CoV‐2 via nasal inoculation, recording weight changes daily for 8 days post‐infection (**Figure** **6**A). The LNP‐nsRNA group showed no difference from the PBS group, with the weight showing a gradual decrease and reached the lowest level of ≈20% decline on the seventh day. However, the weight of mice injected with LNP‐siUSP33 reached its lowest level on the third or fourth day post‐infection, dropping by ≈5%, and gradually returned to its original body weight (Figure 6B). Protein detection showed successful expression of the human ACE2 protein, and USP33 was knocked down in mouse lungs (Figure 6C). The viral load in the lungs of mice in the LNP‐siUSP33 group was significantly lower than that in the PBS and LNP‐nsRNA groups (Figure 6C–E). Immunohistochemistry results were consistent with this, with the viral load significantly reduced in mice injected with LNP‐siUSP33 (Figure 6F). H&E staining showed that LNP‐nsRNA did not alleviate the inflammation caused by viral infection, whereas LNP‐siUSP33 significantly reduced lung inflammatory infiltration (Figure 6G). The detection of inflammatory factors in lung tissues also confirmed the inhibitory effect of LNP‐siUSP33 on viral virulence (Figure 6H). K18‐hACE2 transgenic mice were also used to measure the effect of LNP‐siUSP33 (Figure 6I). Unlike AAV transduction that expressed hACE2 only in the lungs, K18‐hACE2 mice can express hACE2 in multiple organs (Figure S14A,B, Supporting Information). LNP‐siUSP33 significantly prolonged the survival time of K18‐hACE2 mice after infection (Figure S14C, Supporting Information). Reduction of viral load and inflammatory infiltration was obviously observed in the lung tissues of mice by treatment with LNP‐siUSP33 on day 4 post‐infection, but no significant change in viral load was detected in the livers and kidneys because of targeted delivery (Figure 6J–M; Figure S14D,E, Supporting Information). These results strongly indicate that LNP‐siUSP33 can specifically target the lungs and inhibit viral replication, as well as the inflammatory response caused by the virus.

## 3 Discussion

**Figure 7:**
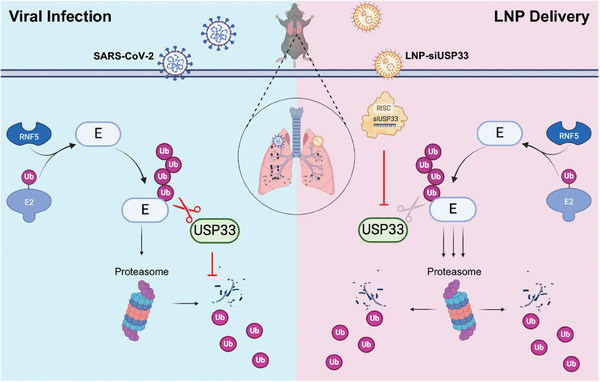
The envelope (E) protein of SARS‐CoV‐2 hijacks the deubiquitinase ubiquitin‐specific protease (USP33) in host cells to cleave off K48‐typed polyubiquitin chains and antagonize E3 ubiquitin ligase RNF5‐mediated degradation. Lipid nanoparticles target the delivery of siUSP33 to the lungs, effectively knock down USP33, and promote the proteasome‐dependent degradation of the E protein, inhibiting viral replication and attenuating virulence.

As an essential component of viral protein particles, E protein plays a crucial role in viral replication and pathogenicity, making it a key drug target. E protein is a relatively unstable protein that can be recognized by the host E3 ubiquitin ligase RNF5 and is rapidly degraded via the proteasome. SARS‐CoV‐2 has also evolved antagonistic strategies to counteract the degradation of viral proteins by hijacking host deubiquitinases. Our study revealed that USP33 was a Dub that selectively targeted E proteins and attenuated RNF5‐induced ubiquitinated degradation. The absence of USP33 resulted in rapid ubiquitination and degradation of the E protein, leading to attenuated viral replication and inflammatory responses. More importantly, we successfully delivered siRNAs targeting USP33 to the lungs of mice using LNPs, which effectively attenuated viral replication and virulence in a mouse infection model (**Figure** **7**).

The E protein was initially found to be susceptible to degradation by the ubiquitin‐proteasome system. However, compared to rapidly degrading viral proteins, such as ORF9b and ORF6, the E protein has a relatively longer half life of ≈16 h. This suggests that the antagonistic effects of Dubs in host cells counteract the degradation of the E protein. According to previous experience, the stability of many proteins can be regulated by multiple deubiquitinases, such as PTEN^[^ ^61^, ^62^ ^]^ and p53.^[^ ^63^, ^64^ ^]^ In comparison to the previously reported USP39, which enhances E protein stability,^[^ ^53^ ^]^ we found that USP33 enhanced E protein stability and promote viral replication more significantly. In contrast to USP39, which showed a slightly up‐regulated expression level after viral infection, we did not see changes in the total protein level of USP33 after viral infection in a variety of cell lines. However, the transcriptomic data from whole blood showed higher mRNA levels of USP33 in patients who developed mild and severe disease after infection compared to the healthy individuals. This indicates that the high expression of USP33 is one of the causes of more severe disease rather than a result of infection. USP33 is usually expressed in both the cytoplasm and the nucleus, and interacts with E protein in the cytoplasm. Interestingly, we found that USP33 in the nucleus migrated to the cytoplasm with SARS‐CoV‐2 infection. However, ectopic expression of the E protein alone had no effect on USP33 localization. This might be due to the presence of other viral proteins that interact with USP33, such as M and ORF7b, which affect its nuclear localization. Based on our results and previous reports, the E protein is mainly localized in the cytoplasm, which reflects that USP33 regulates E protein stability by a different mechanism from that of USP39. These results indicate that the ubiquitination of viral proteins in host cells is complex and multifaceted, highlighting the potential of combination therapeutic strategies.

As the largest family of Dubs, the USP family members typically contain a conserved catalytic domain known as the USP domain, which harbors an active site and is essential for their Dub activity. Currently, apart from a few extensively studied Dubs, such as USP7, there are no specific inhibitors targeting each USP protein, which partly limits the clinical application of Dubs as targets.^[^ ^65^, ^66^, ^67^ ^]^ Based on specificity and targeting, we adjusted the molar ratio of ionizable cationic lipids by adding DOTAP to deliver siUSP33 specifically to the lungs, the main target organ of SARS‐CoV‐2, without affecting the USP33 proteins in other major organs.^[^ ^68^, ^69^, ^70^ ^]^ Furthermore, siUSP33 delivered by LNPs stably knocked down USP33 in the lungs for at least 14 days. As the virus causes acute infections, clinical symptoms in infected patients usually present within 1–3 days of infection, making this strategy sufficient to alleviate most infections and reduce the potential for side effects from prolonged suppression of USP33. This approach provides a novel strategy for the prevention and treatment of COVID‐19.

This study opens avenues for a deeper exploration of the role of USP33 in viral replication and inflammation, providing insights into potential therapeutic targets for combating SARS‐CoV‐2 infections. Future studies should focus on optimizing the delivery system of siUSP33 to enhance its efficacy and minimize off‐target effects. Investigating the broader implications of targeting USP33 in other viral infections may offer valuable insights into the development of broad‐spectrum antiviral strategies.

## 4 Experimental Section

### Cells

The HEK293T, HeLa, A549, and Vero cells were cultured in Dulbecco's modified Eagle's medium (DMEM) (Gibco), Calu3 cells were cultured in MEM (Gibco). HeLa and A549 cells overexpressed human ACE2 protein (HeLa‐ACE2 and A549‐ACE2) using lentiviral transduction and were maintained in DMEM. NHBE cells were purchased form ATCC and grown in DMEM. The mouse‐derived cells including LLC(Cl‐0140), AML12(Cl‐0602) were purchased from Procell and cultured in DMEM. MH‐S cells were cultured in RPMI, TC‐1 cells were cultured in MEM, MEF and NIH‐3T3 were cultured in DMEM, and MLE‐12 were cultured in the specific medium (DMEM+ITS+10 nM Hydrocortisone+10 nMβ‐estradiol). The MLE‐12 cells were supplemented with 2% fetal bovine serum (FBS) (TransGen Biotech) and the other cell lines were supplemented with 10% FBS, as well as penicillin (100 U mL⁻^1^) and streptomycin (100 µg mL⁻^1^). All the cells were cultured at 37 °C with 5% CO_2_.

### Virus and Infection

The SARS‐CoV‐2 WT strain was isolated from nasopharyngeal aspirate specimens sourced from COVID‐19 patients at Shenzhen Third People's Hospital and propagated in Vero E6 cells. Virus infection was performed as previously described. Briefly, when the cell confluence reached 90%, the medium was replaced with serum‐free medium containing the virus. After incubation at 37 °C for 1 h the medium was discarded, and the cells were rinsed with PBS and cultured in medium containing 10% FBS. The medium containing progeny virus was collected and diluted in a threefold gradient, infected with Vero‐E6 cells for 1 h and then replaced with medium containing 1.2% CMC. 24 h later, the cells were fixed with 4% paraformaldehyde and incubated with anti‐N‐HRP antibody, and then stained with KPL to calculate the number of FFUs.

### Plasmids and siRNAs

The expressing plasmids containing SARS‐CoV‐2 genes and different mutant Ubs were constructed in previous work. The SARS‐CoV‐2 E with different tags was inserted into the pCAGGS vector. Flag‐USP33, Flag‐RNF5, Flag‐USP32, and Flag‐USP39 were purchased from SinoBiological, and USP33 was then cloned into pCAGGS or pLVX with different tags. All the genes of the Dub library are listed in Table S1 (Supporting Information).

All siRNAs were synthesized by RIBOBIO and the sense strand sequences are shown below.

hUSP33#1: CCCAGUAAUACAACAUUAATT

hUSP33#2: GGAGAAUAGAUGUUCAUAUTT

hUSP33#3: GCUGCAUUCAUCAAGUCAUTT

mUSP33#1: GCAGGAGACAAAGCAUUAUTT

mUSP33#2: GCCGGCUAAUCUGUUCCAATT

mUSP33#3: GCAGAGCCUCAGAAUCUAUTT

mUSP33#4: GCUGAACCUGGCCCUAUUUTT

### Antibodies and Other Reagents

The following antibodies and reagents were used for immunoblotting and immunoprecipitation in this study:

Rabbit anti‐Flag monoclonal antibody (CST, 14 793); Rabbit anti‐HA monoclonal antibody (CST, 3724); Mouse anti‐Strep‐Tag II monoclonal antibody (Abbkine, 8C12); Rabbit anti‐GST monoclonal antibody (CST, 2625); Mouse anti‐His monoclonal antibody (CST, 2366); Rabbit anti‐Myc monoclonal antibody (CST, 13 987); Mouse anti‐β‐actin monoclonal antibody (TransGen Biotech, HC201‐01); Rabbit anti‐α‐Tubulin polyclonal antibody (Proteintech, 11224‐AP); Rabbit anti‐Histone‐H3 polyclonal antibody (Proteintech, 17168‐1AP); Mouse anti‐Ubiquitin monoclonal antibody (SantaCruz, sc‐8017); Rabbit anti‐USP33 polyclonal antibody (Proteintech, 20445‐1‐AP); Rabbit anti‐RNF5 monoclonal antibody (ab308066); Rabbit anti‐SARS‐CoV‐2 E monoclonal antibody (abcam, ab308371); Rabbit anti‐SARS‐CoV‐2‐M monoclonal antibody (abcam, ab308415); Rabbit anti‐SARS‐CoV‐2‐ORF7b monoclonal antibody (abcam, ab313933); Mouse anti‐SARS‐CoV‐2‐N monoclonal antibody (SinoBiological, 40143‐MM05); Rabbit anti‐SARS‐CoV‐2‐S polyclonal antibody (SinoBiological, 40590‐T46); Rabbit anti‐SARS‐CoV‐2 NSP3 monoclonal antibody (CST, 88 086); Rabbit anti‐hACE2 polyclonal antibody (CST, 4355); Goat anti‐Rabbit and anti‐Mouse HRP Conjugated secondary antibodies (TransGen Biotech); Goat anti‐Rabbit Alexa Fluo 488 Conjugated secondary antibodies (Invitrogen, A‐11008); Goat anti‐Mouse Alexa Fluo 555 Conjugated secondary antibodies (Invitrogen, A‐21422); DAPI (Beyotime, C1002); Anti‐Flag magnetic Beads (Sigma, M8823); Anti‐HA magnetic beads (Sigma, SAE0197); Anti‐Myc magnetic beads (Sigma, SAE0201); Protein A/G agarose (GE healthcare); Magnetic Beads‐conjugated Rabbit anti GST‐Tag mAb (ABclonal, AE122); Reduced Glutathione (Solarbio, G8180) and IPTG (Solarbio, I1020).

### Lentiviral Preparation and Stable Cell Line Construction

Lentivirus production was performed in HEK293T cells. Briefly, the lentiviral packaging plasmids and shRNA were co‐transfected into cells at a ratio of 5:7:10 (psPAX2: pMD2.G: shRNA). After 48 h of transfection, the culture supernatant was collected, centrifuged, and filtered through a 0.45 µm filter M to obtain purified viral particles. The purified lentivirus was used to infect HEK293T, A549, Calu3, and HeLa cells in the presence of polybrene (5 µg mL⁻^1^) at 37 °C by centrifugation at 1800 rpm for 40 min. After 48 h of infection, puromycin (1–2 µg mL⁻^1^) was added to the medium to select cells infected with the lentivirus. Immunoblotting with specific antibodies was performed to assess the efficiency of target protein knockdown or overexpression. All the shRNA and sgRNA sequences are listed in Table S2 (Supporting Information).

### RNA Isolation and Quantitative Real‐Time PCR

TRIzol reagent was used to isolate total RNA according to the manufacturer's instructions. The RNA concentration and purity were assessed using a spectrophotometer. Subsequently, cDNA was synthesized from the extracted RNA using a reverse transcription kit. Quantitative real‐time PCR (qPCR) was performed to analyze the expression levels of target genes. The qPCR reactions were set up with specific primers, cDNA templates, and a SYBR Green master mix. The amplification and quantification of target gene expression were carried out using a real‐time PCR machine, and the results were analyzed based on the Ct values. All human and mouse qRT‐PCR primers used in this study are listed in Table S3 (Supporting Information).

### Protein Interaction Prediction by AlphaFold 3

The interaction of USP33 and E was predicted with the current most accurate AlphaFold 3. In order to improve accuracy, the zinc finger and the disorder domain of USP33 (185–716 aa kept) were first removed, then the whole E and simplified USP33 sequence were submitted to AlphaFold Server (https://golgi.sandbox.google.com/). The optimal predicted complex structure was selected by considering the pLDDT score (>70), PAE plot and pTM (>0.5) scores. Finally, the interaction residues between USP33 and E were calculated and visualized with Pymol (Version 2.5.0 Open‐Source).

### In Vivo and In Vitro Ubiquitination Assay

The ubiquitination assay was carried out as previously described.^[^ ^19^, ^20^ ^]^ For in vivo ubiquitination assay, the HEK293T cells expressing target proteins were sonicated in lysis buffer supplemented with 0.5% NP‐40 and 1% SDS and then denatured at 95 °C for 5 min. The cell lysates were diluted with 900 µL of SDS‐free lysis buffer, of which 1/10 was saved as WCL for detecting the levels of target proteins. The remaining cell lysates were immunoprecipitated by antibodies of indicated proteins, followed by protein A/G. Immunoprecipitated proteins were washed three times by lysis buffer and subjected to immunoblotting analysis.

For in vitro ubiquitination assay, the E gene of SARS‐CoV‐2 was inserted into the pGEX plasmid and expression was induced in BL21(DE3) by IPTG. The GST‐E protein was enriched by glutathione Sepharose beads. In addition, the E3 ubiquitin ligase RNF5, Deubiquitinase USP33‐WT, and USP33‐Mu were overexpressed in HEK293T cells and precipitated by Flag‐beads, which were then eluted by Flag‐peptide. The beads with GST‐E protein were incubated with USP33‐WT or Mu, together with 200 ng of E1 (UBE1; abcam), 400 ng of E2 (UBE2K; abcam), 2 µg of recombinant ubiquitin (abcam), and 1 µg RNF5 in reaction buffer (50 mM Tris [PH 7.4], 2 mM MgCl_2_, 4 mM ATP [Sigma]) at 37 °C for 1 h. The supernatant was removed, and the reaction was terminated by adding 2 × protein loading buffer. The ubiquitin chain was detected by immunoblotting.

### Half‐Life Analysis

The half life analysis of target proteins was performed as previously described.^[^ ^19^ ^]^ Briefly, the cells expressing E protein were equally divided into 12‐well plates. When the cell confluence reached 90%, CHX (Sigma, 50 µg mL⁻^1^) was added into the cells of all wells at the same time, and cells were collected at different time points to detect the level of E protein. The proteasome or lysosome inhibitors were added into the cells at the same time as CHX.

### Preparation of mRNA‐LNPs

Selective organ targeting nanotechnology was incorporated into the traditional MC3‐based LNP formulation used by the FDA‐approved RNAi therapy named Patisiran/Onpattro by adding the supplementary cationic lipids DOTAP (1,2‐dioleoyl‐3‐trimethylammonium‐propane). This generated highly lung‐selective LNPs with a final molar ratio of Dlin‐MC3‐DMA:DSPC (1,2‐distearoyl‐*sn*‐glycero‐3‐phosphocholine):cholesterol:DMG‐PEG:DOTAP at 25:5:19.3:0.8:50. LNP‐siNC and LNP‐siRNA had high reproducibility and were ≈100 nm in size, spherical and evenly distributed, as determined by Zetasizer and transmission electron microscopy.

### Cryogenic Transmission Electron Microscopy (Cryo‐TEM)

The Cryo‐TEM sample preparation and imaging procedures were conducted in the following manner: First, a volume of 5 µL of the sample was meticulously applied onto a glow‐discharged grid (R1.2/1.3 Au, 300 mesh, GiG). Subsequently, the grids were subjected to blotting for a duration of 4 s under 100% humidity at a temperature of 4 °C. Without delay, the grids were rapidly submerged into liquid ethane by employing a Mark IV vitrobot (ThermoFisher). The imaging process was performed utilizing a Talos Glacios transmission electron microscope (ThermoFisher). The magnification level was set to 92000 ×, with a pixel size of 1.57 Å.

### In Vivo Imaging Software Imaging

LNPs encapsulating CY5 modified siRNA were injected into C57BL/6 mice intravenously through the retro‐orbital route (0.5 mg  kg⁻^1^). Whole body and major organs (heart, liver, spleen, lungs, kidney) were then imaged for fluorescence using an IVIS Lumina XR system with Living Image Software v.4.3.1 (Caliper Life Science).

### Toxicity Profiling

C57BL/6 mice were used for in vivo toxicity studies. Mice were injected through Retro‐orbital vein with LNP‐siUSP33, LNP‐nsRNA or PBS, respectively. Each group had three mice. Blood was collected one day or four days after the injection. the account of the WBC(white blood cell), RBC(Red blood cell), HGB (hemoglobin), and PLT(Platelet) was assessed by the Mira cell fluorescence analyzer. And the Automatic Biochemical Analyzer Chemray‐240 is utilized to measure the levels of ALT (glutamic‐pyruvic transaminase), AST (glutamic oxalacetic transaminase), ALP (Alkaline phosphatase), and TBA (Total bile acid), UREA (blood urea), CREA (creatinine), and UA (blood uric acid), which serve as biochemical indicators of liver and kidney cell injury.

### Peripheral Blood Transcriptomics

C57BL/6 mice were used for in vivo toxicity studies. Mice were injected with LNP‐siUSP33, LNP‐nsRNA, or PBS via the retro‐orbital route, respectively. Each group had three mice and the blood was collected on day 1 and day 4 after the injection. RNA was extracted using the TRIzol reagent (Invitrogen, Carlsbad, CA, USA). And performed the 2 × 150 bp paired‐end sequencing on an illumina Novaseq 6000 (LC‐Bio Technology CO., China). Then, the ​fastp software (https://github.com/OpenGene/fastp) was used to remove the miscorrect reads. After that, Ensembl was used to map reads to the reference genome of mus musculus Ensembl_v107. All transcriptomes were merged to reconstruct a comprehensive transcriptome using gffcompare (https://github.com/gpertea/gffcompare/). After the final transcriptome was generated, StringTie (https://ccb.jhu.edu/software/stringtie) was used to perform expression level of mRNAs by calculating FPKM. The differentially expressed mRNAs were selected by R package edgeR. The Peripheral Blood Transcriptomics are available from the Gene Expression Omnibus (http://www.ncbi.nlm.nih.gov/geo/) with the GEO accession GSE277027. All the identified genes were shown in the Supporting Information as Table S4 (Supporting Information).

### Animal Experiments

C57BL/6 mice aged 6 to 8 weeks were intranasally transduced with 5 × 10^11^ GC AAV‐hACE2. 6 days later, mice were given PBS, LNP‐nsRNA, and LNP‐siUSP33 by Retro‐orbital vein. One day after the injection treatment, mice were anesthetized and intranasally inoculated with 1 × 10^5^ FFU WT SARS‐CoV‐2 strain. The body weights of the mice were monitored daily, and their lungs were harvested for the analysis of the viral titer, mRNA abundance, Western blotting, Immunofluorescence and H&E staining. All the animal experiments related to SARS‐CoV‐2 were conducted in the ABSL‐3 Laboratory of Shenzhen Third People's Hospital.

### H&E Staining and Immunohistochemistry Analysis

Lungs from infected and non‐infected mice were dissected out in ABSL‐3 and initially fixed in 4% paraformaldehyde for 24 h. The samples were then dehydrated, and subsequently embedded in paraffin. Thin sections (4–5 µm) were cut and stained with Hematoxylin and Eosin for visualization of tissue morphology. For immunohistochemistry, paraffin‐embedded sections were deparaffinized, dehydrated, and subjected to antigen retrieval. Blocking proteins preceded the incubation with primary antibodies specific to SARS‐CoV‐2‐Nucleoprotein, followed by incubation with HRP labeled secondary antibodies and DAB for visualization of antigen localization and expression levels in the tissue.

### Statistical Analysis

All results are shown as the mean ± SD. Student's t test (unpaired, two‐tailed) was used to compare two independent groups while two‐way analysis of variance (ANOVA) was performed for comparisons of multiple groups. All statistical analyses were performed using GraphPad Prism. *P* values < 0.05 were considered statistically significant. All experiments were repeated three times or more.

### Ethics

The animal experiments related to authentic SARS‐CoV‐2 were conducted in the ABSL‐3 Laboratory of Shenzhen Third People's Hospital. The animal experimental procedures used in the study were approved by the Institutional Animal Care and Use Committee of Shenzhen Third People's Hospital (NO. 2023‐015). The procedures of animal imaging experiments followed the ethical guidelines approved by the Department of laboratory Animals, Central South University (CSU‐2024‐0100).

## Conflict of Interest

The authors declare no conflict of interest.

## Author Contributions

Y.Z., Y.L., and L.F. contributed equally to this work. Y.Z., Y.L., X.W., and L.F. performed laboratory experiments. Y.Z., Y.L., W.F., and C.Y. analyzed bioinformatics data. Y.Z., X.W., and X.G. performed the viral infection. Q.H. predicted the interactions using AlphaFold 3. Y.Z. and Y.L. prepared the figures. Y.W., X.S., and J.Z. made the statistical analysis. Z.Z., Z.X., and Y.Z. supervised the research. Y.Z. and Y.L. wrote the manuscript. Z.Z. and Z.X. conceived the project. All authors have read and approved the article.

## Supporting information



Supporting Information

Supporting Information

## Data Availability

The data that support the findings of this study are available from the corresponding author upon reasonable request.
