# Supplementary material for: Lung‐Targeted Lipid Nanoparticle‐Delivered siUSP33 Attenuates SARS‐CoV‐2 Replication and Virulence by Promoting Envelope Degradation
Source: Adv Sci (Weinh). 2024 Sep 20;11(42):2406211. doi: 10.1002/advs.202406211 (PMC11558077; doi:10.1002/advs.202406211)
Supplement: Supplementary file 1 — Supporting Information [file ADVS-11-2406211-s002.docx]

**Lung-targeted lipid nanoparticle-delivered siUSP33 attenuates SARS-CoV-2 replication and virulence by promoting envelope degradation**

Yuzheng Zhou^1#^, Yujie Liao^2#^, Lujie Fan^1,3#^, Xiafei Wei^1^, Qiang Huang^1^, Chuwei Yang^1^, Wei Feng^1^, Yezi Wu^1^, Xiang Gao^1^, Xiaotong Shen^1^, Jian Zhou^1^, Zanxian Xia^2,4*^, Zheng Zhang^1,5*^

^1^Institute for Hepatology, National Clinical Research Center for Infectious Disease, Shenzhen Third People's Hospital, The Second Affiliated Hospital, School of Medicine, Southern University of Science and Technology, Shenzhen, China.

^2^Department of Cell Biology, School of Life Sciences, Central South University, Changsha, China.

^3^Guangzhou Laboratory, Guangzhou, China.

^4^Hunan Key Laboratory of Animal Models for Human Diseases, Hunan Key Laboratory of Medical Genetics & Center for Medical Genetics, School of Life Sciences, Central South University, Changsha, China.

^5^Shenzhen Research Center for Communicable Disease Diagnosis and Treatment, Chinese Academy of Medical Sciences, Shenzhen, China.

**^#^**These authors contribute equally.

*Correspondence: Zheng Zhang, [zhangzheng1975@aliyun.com](mailto:zhangzheng1975@aliyun.com);

Zanxian Xia, xiazanxian@sklmg.edu.cn

**This file includes:**

Figures S1 to S14

Tables S1 to S3

**Supplementary Figures**

**Figure S1**

**
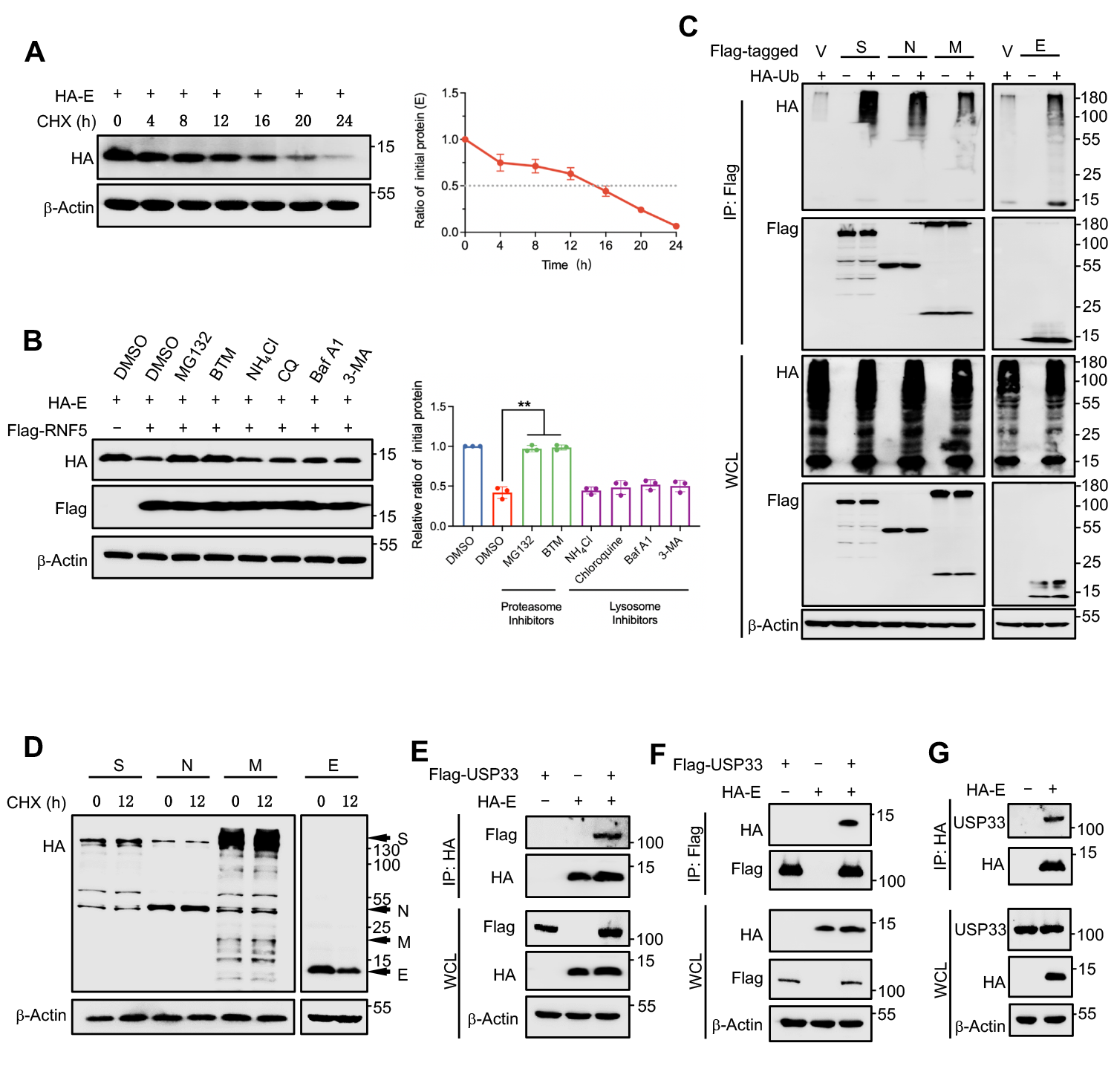
**

**Figure S1. Dubs screening identified USP33 as an important factor in promoting viral replication by targeting E (related to Figure 1).**

**(A).** HEK293T cells transfected with plasmids expressing HA-E were treated with 50 μg/ml cycloheximide (CHX), and collected at indicated time to detect the protein levels of E by immunoblotting (left). Quantification was shown as mean ± SD (n=3 independent experiments) (right)

**(B).** HEK293T cells were co-transfected with HA-E and Flag-RNF5, and then treated with indicated inhibitors for 8 h before collection. The protein levels of E were analyzed by immunoblotting (left) and quantification was shown as mean ± SD (n=3 independent experiments) (right). Student’s *t* *t*est (unpaired, two-tailed), ***P*<0.01.

**(C).** The Flag-tagged viral genes and HA-Ub were co-transfected into HEK293T cells. The WCLs were denatured and then precipitated by anti-Flag beads, and analyzed by immunoblotting with indicated antibodies.

**(D).** HEK293T cells expressing different viral proteins were treated with CHX for indicated time and analyzed by immunoblotting to detect the protein levels.

**(E-F).** HEK293T cells were co-transfected with Flag-USP33 and HA-E, followed by immunoprecipitation with anti-HA (E) or anti-Flag (F) beads. The WCLs and precipitated proteins were analyzed by immunoblotting.

**(G).** Empty vector or HA-E were transfected into HEK293T cells, followed by immunoprecipitation with anti-HA beads. The E and endogenous USP33 were detected by anti-HA and anti-USP33 antibodies.

**Figure S2**

**
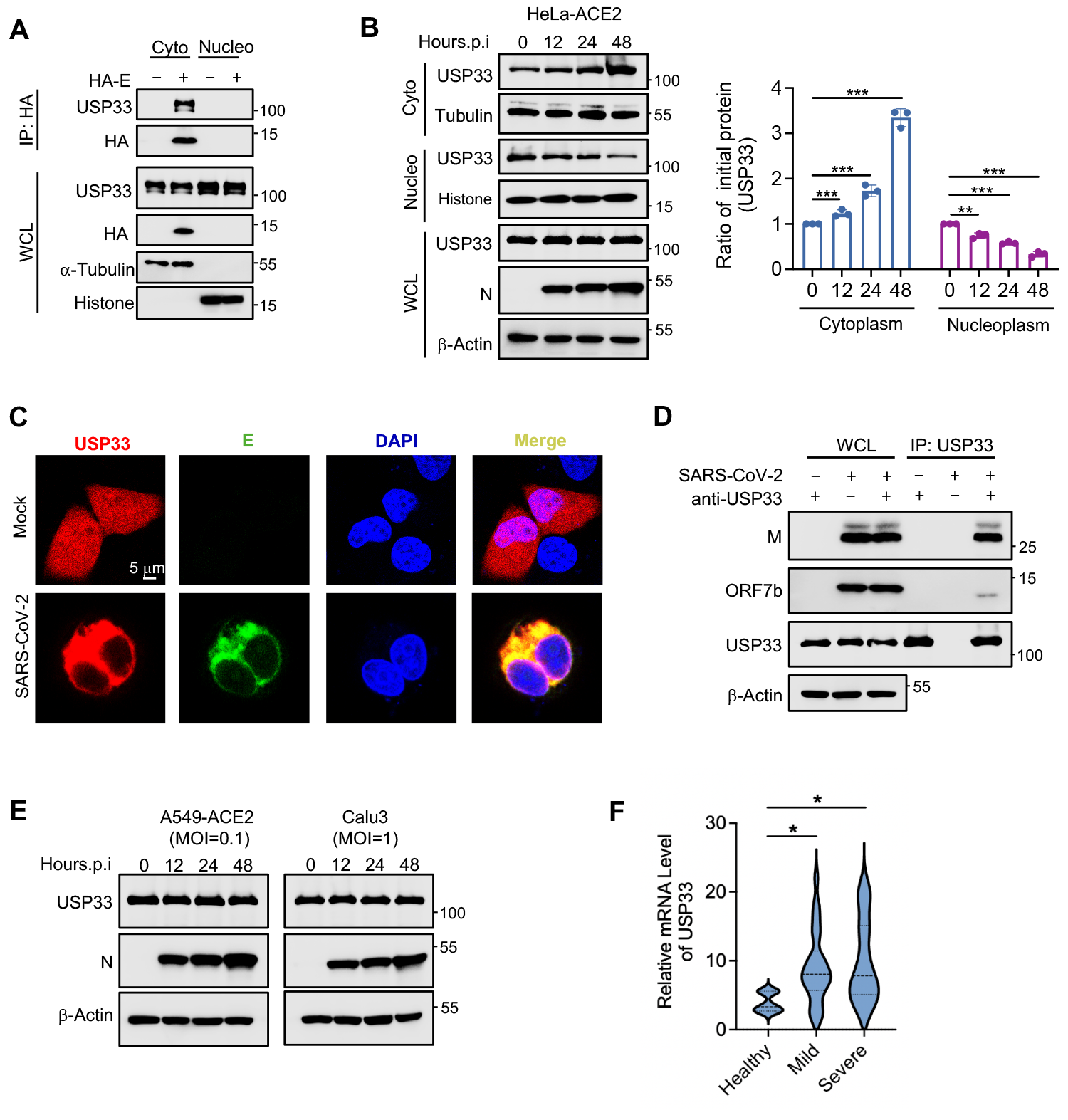
**

**Figure S2. SARS-CoV-2 changes USP33 localization without affecting protein levels (related to Figure 1).**

**(A).** HEK293T cells transfected with HA-E were collected for cytoplasmic and nuclear protein extraction. Both cytoplasm and nucleoplasm were immunoprecipitated by anti-HA beads and then analyzed via immunoblotting.

**(B).** HeLa-ACE2 cells infected with SARS-CoV-2 at a MOI of 0.1 were collected at indicated time for cytoplasmic and nuclear protein extraction, followed by immunoblotting to detect the USP33 (left). Quantification was shown as mean ± SD (n=3 independent experiments) (right). Student’s *t* *t*est (unpaired, two-tailed), ***P*<0.01, ****P*<0.001.

**(C).** HeLa-ACE2 cells were infected with SARS-CoV-2 at a MOI of 0.1 for 24 h, and immunofluorescence was performed with the indicated antibodies. Scale bars, 5 μm.

**(D).** HeLa-ACE2 cells infected with SARS-CoV-2 or not were lysed and immunoprecipitated by anti-USP33. The indicated proteins were detected via immunoblotting.

**(E).** A549-ACE2 and Calu3 cells infected with SARS-CoV-2 were collected at indicated time to detect the protein levels of USP33 using immunoblotting.

**(F).** Changes of the USP33 mRNA levels in the whole blood of patients infected with SARS‐CoV‐2 compared with that in healthy individuals were depicted using data from the public data set HRA002996 of GEO database. Statistical significance was determined using an unpaired, two‐tailed Student's *t*‐test. **P*<0.05.

**Figure S3**

**
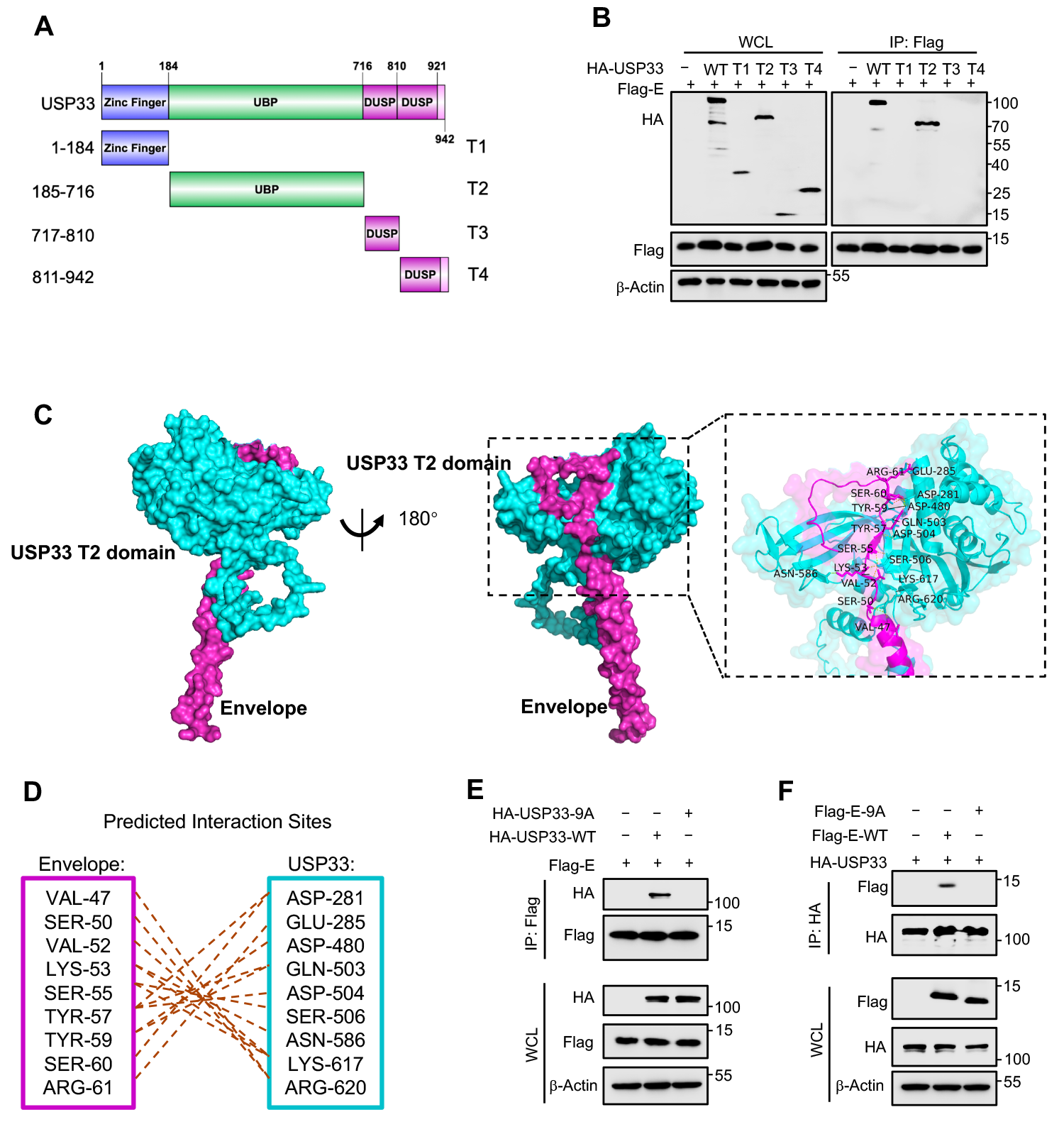
**

**Figure S3. The UBP domain of USP33 interacts with SARS-CoV-2 E protein (related to Figure 1).**

**(A).** A pattern diagram showing the structural domains of USP33.

**(B).** HEK293T cells transfected with indicated plasmids were lysed and immunoprecipitated by anti-Flag beads, and the WCLs as well as precipitated proteins were analyzed via immunoblotting.

**(C).** A structural model of the “USP33-E” complex was presented in two orthogonal views. The model was generated using AlphaFold 3. SARS-CoV-2 E protein (magenta), USP33-T2 domain (cyan).

**(D).** The predicted binding sites of USP33 and E proteins.

**(E-F).** HEK293T cells transfected with indicated plasmids were lysed and immunoprecipitated by anti-Flag (E) or anti-HA (F) beads. The WCLs and precipitated proteins were analyzed using immunoblotting.

**Figure S4**

**
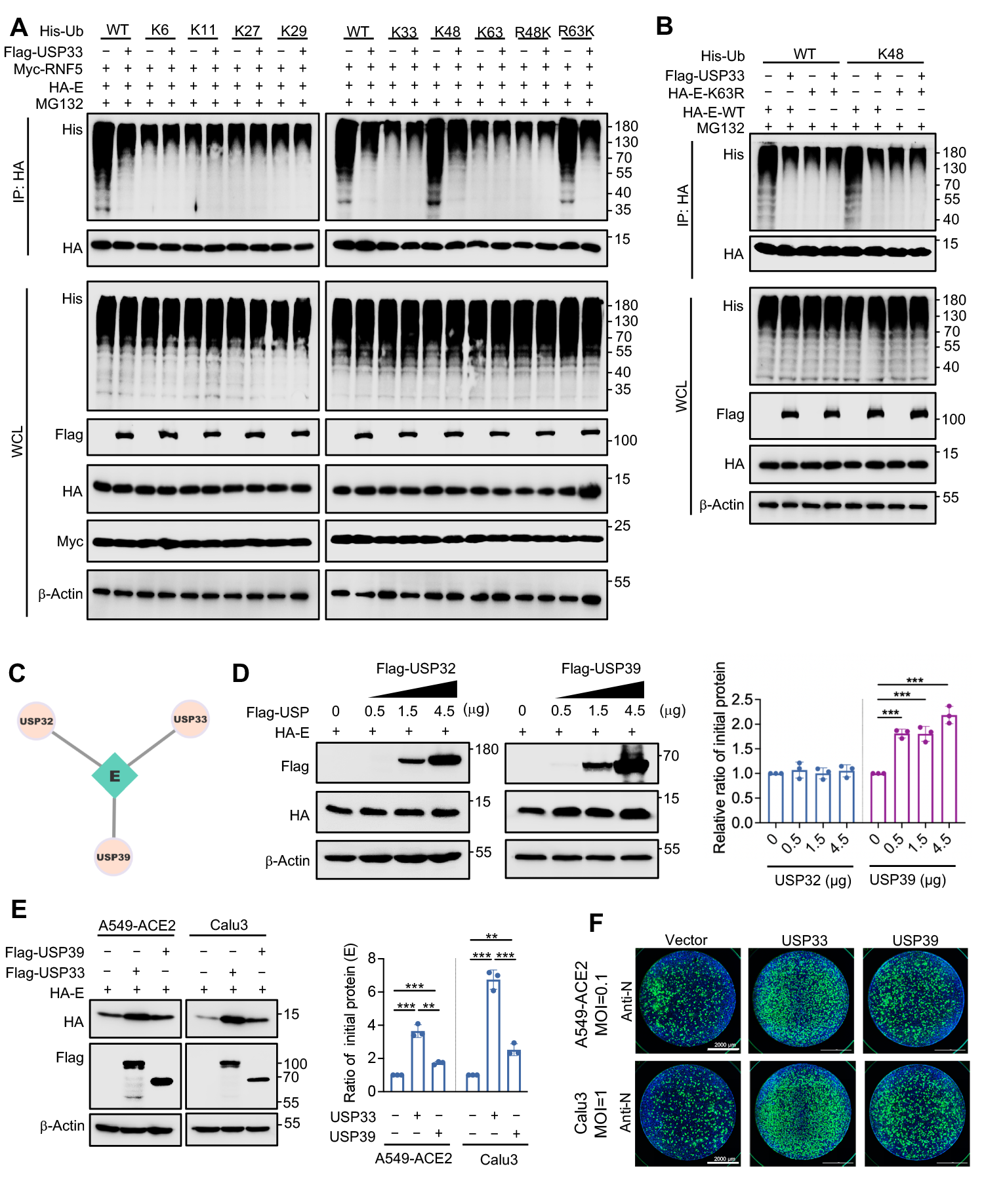
**

**Figure S4. USP33 enhances E stabilization by removing K48-typed polyubiquitin chains (related to Figure 2).**

**(A).** The plasmids containing HA-E, Myc-RNF5, Flag-USP33 and different mutant types of His-Ub were co-transfected into HEK293T cells and the cells were treated with MG132. The WCLs were denatured and immunoprecipitated with anti-HA beads, followed by immunoblotting.

(B). HEK293T cells were transfected with indicated plasmids and treated with MG132. The WCLs were denatured and immunoprecipitated with anti-HA beads, followed by immunoblotting.

**(C).** Protein interaction maps based on published databases showing Dubs that potentially bind to E protein.

**(D).** HEK293T cells expressing HA-E were transfected with increasing amount of plasmids containing Flag-USP32 or Flag-USP39. The cells were lysed to detect the protein level of E by immunoblotting (left). Quantification was shown as mean ± SD (n=3 independent experiments) (right). Student’s *t* *t*est (unpaired, two-tailed), ****P*<0.001.

**(E).** A549-ACE2 and Calu3 cells expressing HA-E were transfected with Flag-USP33 or Flag-USP39. The cells were collected for detecting USP33 protein levels using immunoblotting (left). Quantification was shown as mean ± SD (n=3 independent experiments) (right). Student’s *t* *t*est (unpaired, two-tailed), ***P*<0.01, ****P*<0.001.

**(F).** A549-ACE2 and Calu3 cells expressing Flag-USP33 or Flag-USP39 were infected with SARS-CoV-2 for 24h. The relative ratio of infection was analyzed via immunofluorescence.

**Figure S5**

**
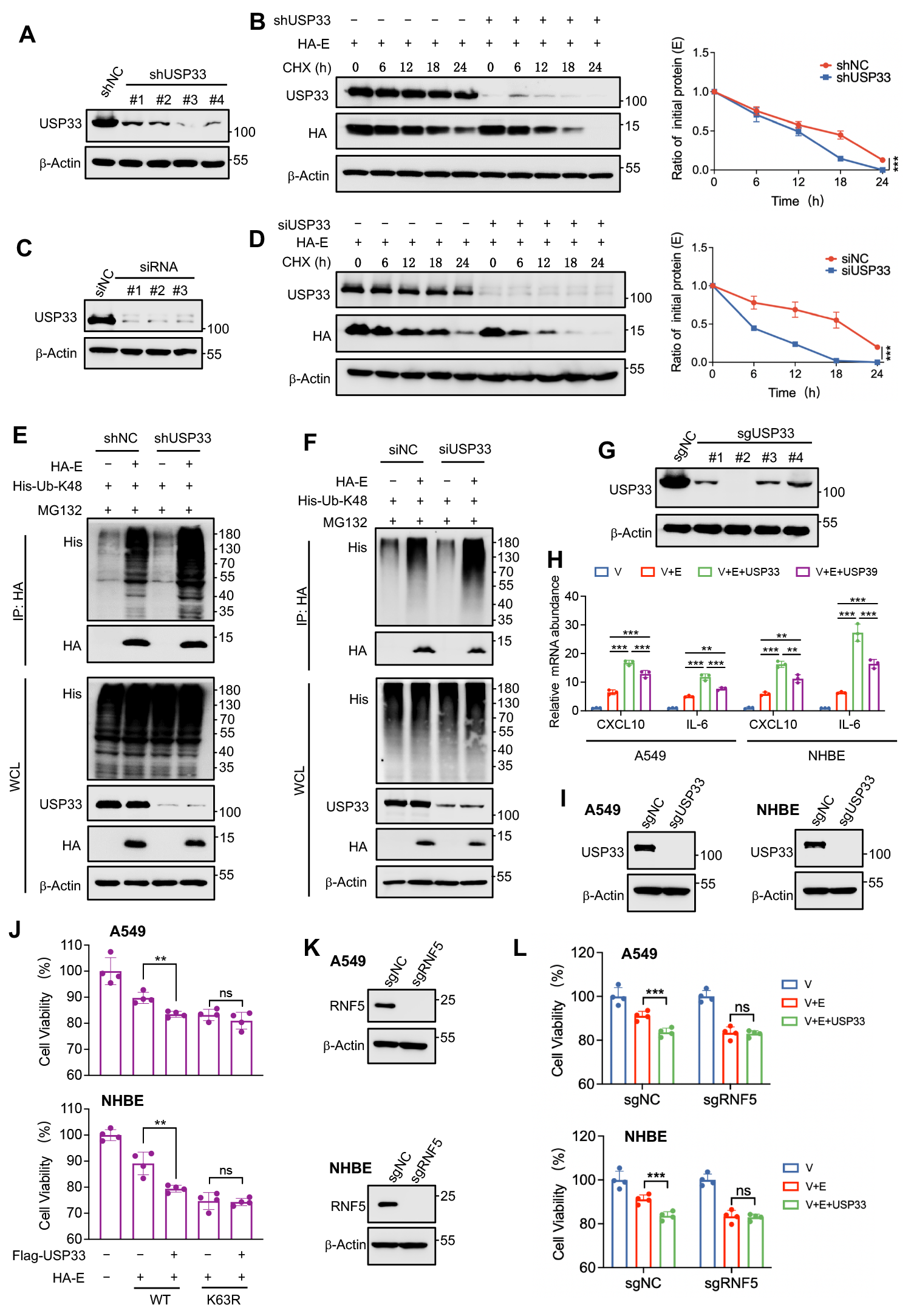
**

**Figure S5. USP33 enhances the ability of E proteins to induce inflammatory response and cell death (related to Figure 2).**

**(A).** Four shRNAs targeting USP33 and shNC were packaged into the lentivirus and transduced into HEK293T cells, respectively. The USP33 protein levels was detected by immunoblotting.

**(B).** The plasmids containing HA-E were transfected into HEK293T cells stably expressing shNC or shUSP33. The cells were treated with CHX for indicated time and collected for immunoblotting to analyze the protein level of E (left). Quantification of E protein relative to β-Actin was shown (right).

**(C).** Three siRNAs targeting USP33 and siNC were transfected into HEK293T cells for 48h. The USP33 protein levels was detected by immunoblotting.

**(D).** The plasmids expressing HA-E were transfected into HEK293T cells containing siNC or siUSP33. The cells were treated with CHX for indicated time and collected for immunoblotting to analyze the protein level of E (left). Quantification of E protein relative to β-Actin was shown (right).

**(E).** HEK293T cells stably expressing shNC or shUSP33 were transfected with indicated plasmids and treated with MG132. The WCLs were denatured and immunoprecipitated with anti-HA beads for immunoblotting.

**(F).** The polyubiquitinated chains assay of HA-E in HEK293T cells transfected with siNC or siUSP33.

**(G).** Four sgRNAs targeting USP33 and sgNC were packaged into the lentivirus and transduced into HEK293T cells, followed by treatment with puromycin(2 μg/ml) for 48h. The USP33 protein levels was detected by immunoblotting.

**(H).** A549 and NHBE cells transfected with indicated plasmids were lysed to extract the total RNA, and the relative mRNA levels of *CXCL10* and *IL6* were analyzed by qRT-PCR.

**(I).** A549 and NHBE cells were transduced with sgUSP33 or sgNC by lentivirus, followed by treatment with puromycin(2 μg/ml) for 48h. The USP33 protein levels were detected by immunoblotting.

**(J).** The cell viability of A549 and NHBE cells transfected with indicated plasmids was assayed using the CCK-8 reagent.

**(K).** A549 and NHBE cells were transduced with sgRNF5 or sgNC by lentivirus, followed by treatment with puromycin(2 μg/ml) for 48h. The RNF5 protein levels were detected by immunoblotting.

**(L).** A549-WT, A549-RNF5-KO, NHBE-WT and NHBE-RNF5-KO cells were transfected with indicated plasmids, and the cell viability was analyzed using the CCK-8 reagent.

Student’s *t*test (unpaired, two-tailed) was used to compare two independent groups, and two-way ANOVA test was performed for comparisons of multiple groups. ***P*<0.01; ****P*<0.001, ns, not significant.

**Figure S6**

**
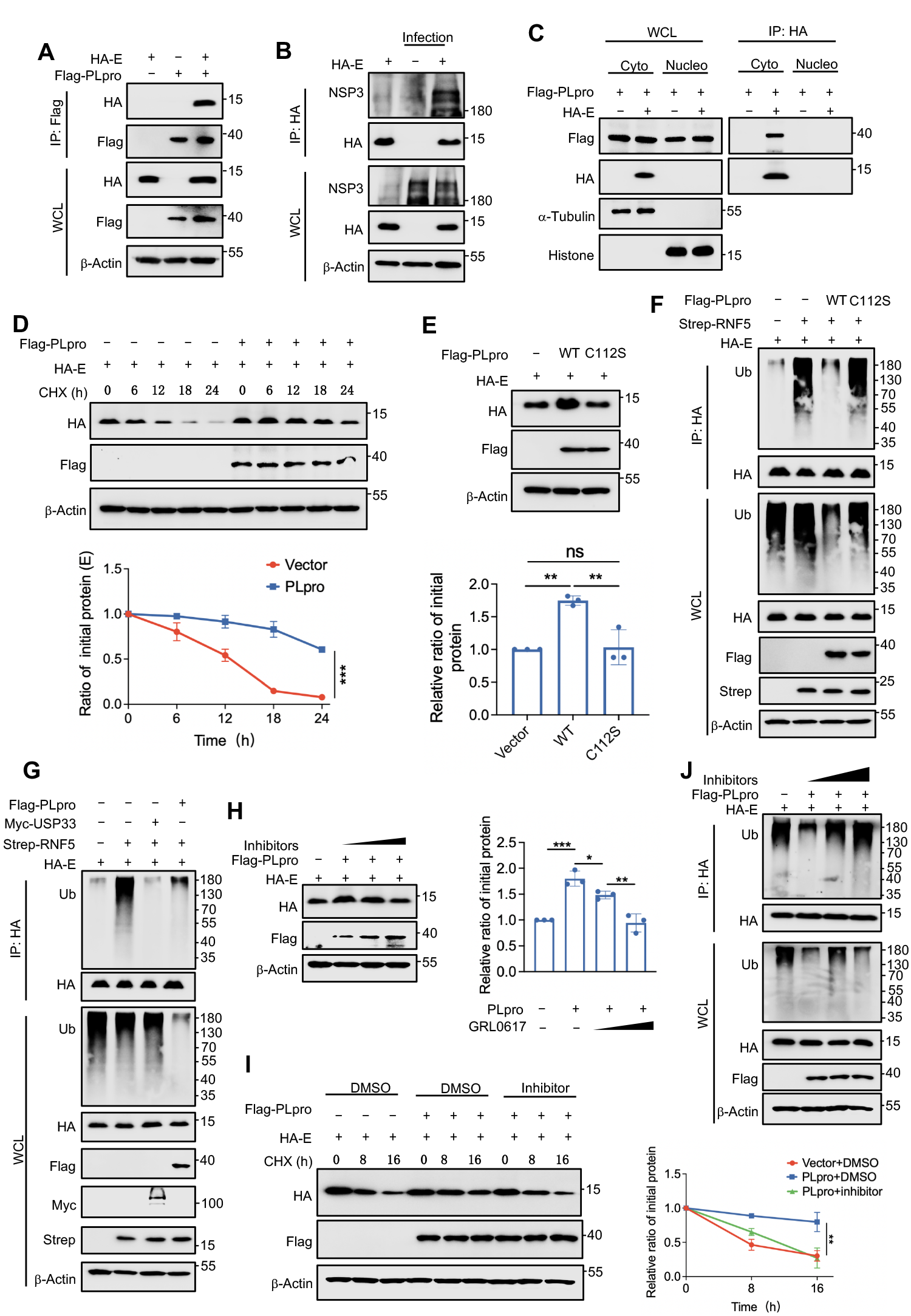
**

**Figure S6. PLpro and USP33 synergize to strengthen E protein stability (related to Figure 2).**

**(A).** HEK293T cells transfected with indicated plasmids were lysed and immunoprecipitated by anti-Flag beads. The WCLs and precipitated proteins were analyzed by immunoblotting.

**(B).** HeLa-ACE2 cells were transfected with plasmids containing HA-E and infected with SARS-CoV-2 at a MOI of 0.1 for 48 h. The WCLs and precipitated proteins were analyzed by immunoblotting.

**(C).** HEK293T cells transfected with HA-E and Flag-PLpro were collected for cytoplasmic and nuclear proteins extraction. Both cytoplasm and nucleoplasm were precipitated by anti-HA beads and then analyzed by immunoblotting.

**(D).** HEK293T cells expressing HA-E were transfected with empty vector or Flag-PLpro, treated with CHX and collected at indicated time for immunoblotting to analyze the protein level of E (up). Quantification relative to β-Actin was shown (down).

**(E).** HEK293T cells expressing HA-E were transfected with empty vector, Flag-PLpro-WT or Flag-PLpro-C112S, and collected for analyzing the E protein level by immunoblotting (up). Quantification relative to β-Actin was shown (down).

**(F-G).** HEK293T cells transfected with indicated plasmids were lysed, denatured and immunoprecipitated by anti-HA beads. The WCLs and precipitated proteins were analyzed by immunoblotting.

**(H).** HEK293T cells expressing HA-E were transfected with plasmids and treated with inhibitors as indicated. The protein levels of E were detected (left) and quantified (right).

**(I).** HEK293T cells expressing HA-E were treated with inhibitors and CHX as indicated. The cells were collected at different time for immunoblotting. Quantification relative to β-Actin was shown (right).

**(J).** HEK293T cells transfected with plasmids and treated with inhibitors as indicated. The cells were lysed, denatured and immunoprecipitated by anti-HA beads, followed by immunoblotting.

Student’s *t*test (unpaired, two-tailed) was used to compare two independent groups, and two-way ANOVA test was performed for comparisons of multiple groups. **P*<0.05; ***P*<0.01; ****P*<0.001.

**Figure S7**

**
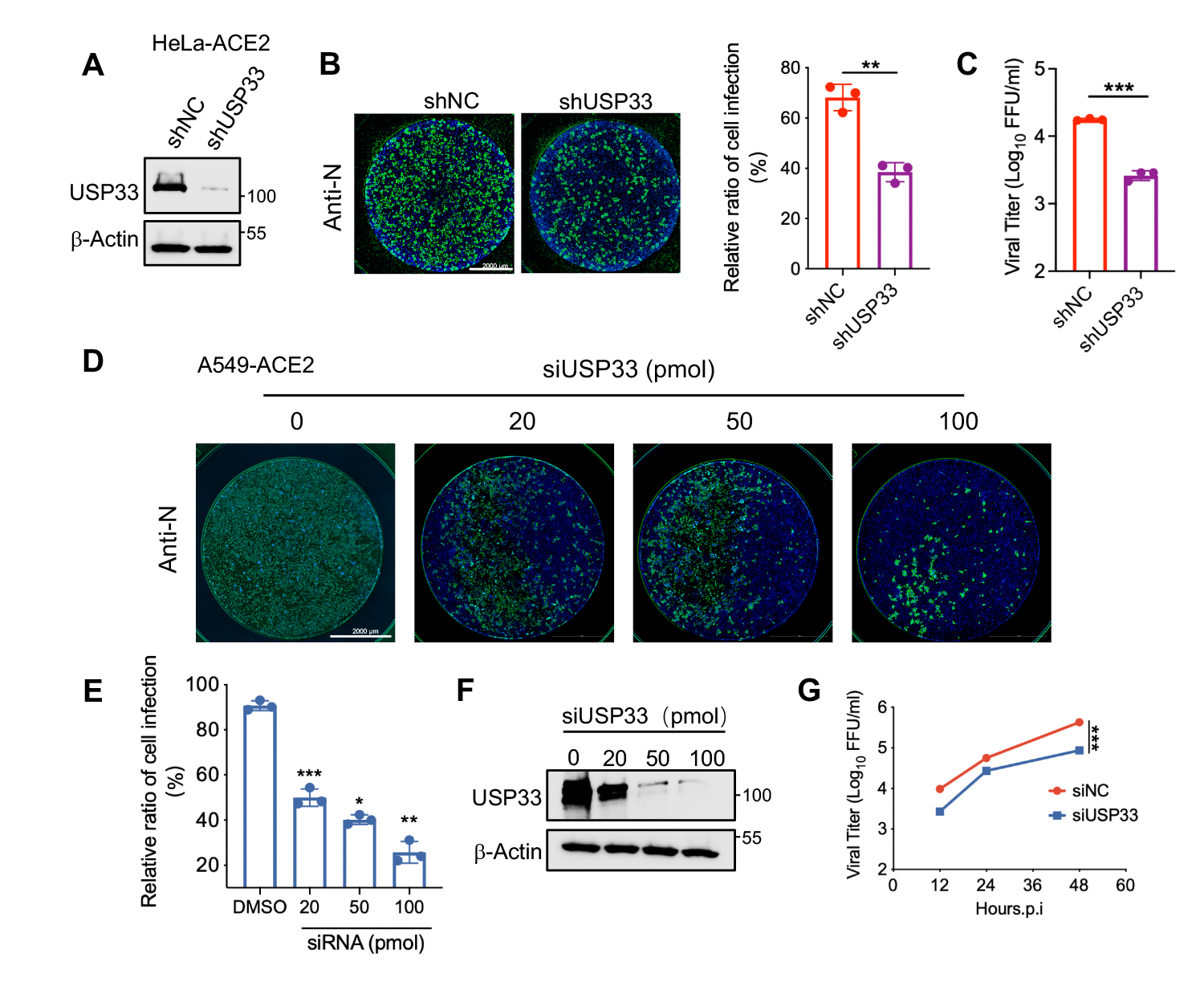
**

**Figure S7. USP33 can facilitate SARS-CoV-2 replication (related to Figure 3).**

**(A).** HeLa-ACE2 cells were transduced with shUSP33 by lentivirus for 48 h. The protein levels of USP33 were analyzed by immunoblotting.

**(B).** USP33 knockdown or WT HeLa-ACE2 cells were infected with SARS-CoV-2 at a MOI of 0.1 for 24 h. The relative ratio of infection was analyzed by immunofluorescence (left) and quantification was shown (right).

**(C).** The viral titers of HeLa-ACE2 cells infected with SARS-CoV-2 at a MOI of 0.1 for 24h were analyzed by FFA and quantified.

**(D-F).** A549-ACE2 cells were transfected with increasing amount siRNAs targeting USP33 and then infected with SARS-CoV-2 at a MOI of 0.1. The relative ratio of infection was analyzed by immunofluorescence (D) and quantified (E), and the protein levels of USP33 were detected by immunoblotting (F).

**(G).** A549-ACE2 cells were transfected with siNC or siUSP33 and infected with SARS-CoV-2 at a MOI of 0.1. The culture media were collected at indicated time to analyze the viral titers by FFA.

Student’s *t*test (unpaired, two-tailed) was used to compare two independent groups, and two-way ANOVA test was performed for comparisons of multiple groups. **P*<0.05; ***P*<0.01; ****P*<0.001.

**Figure S8**

**
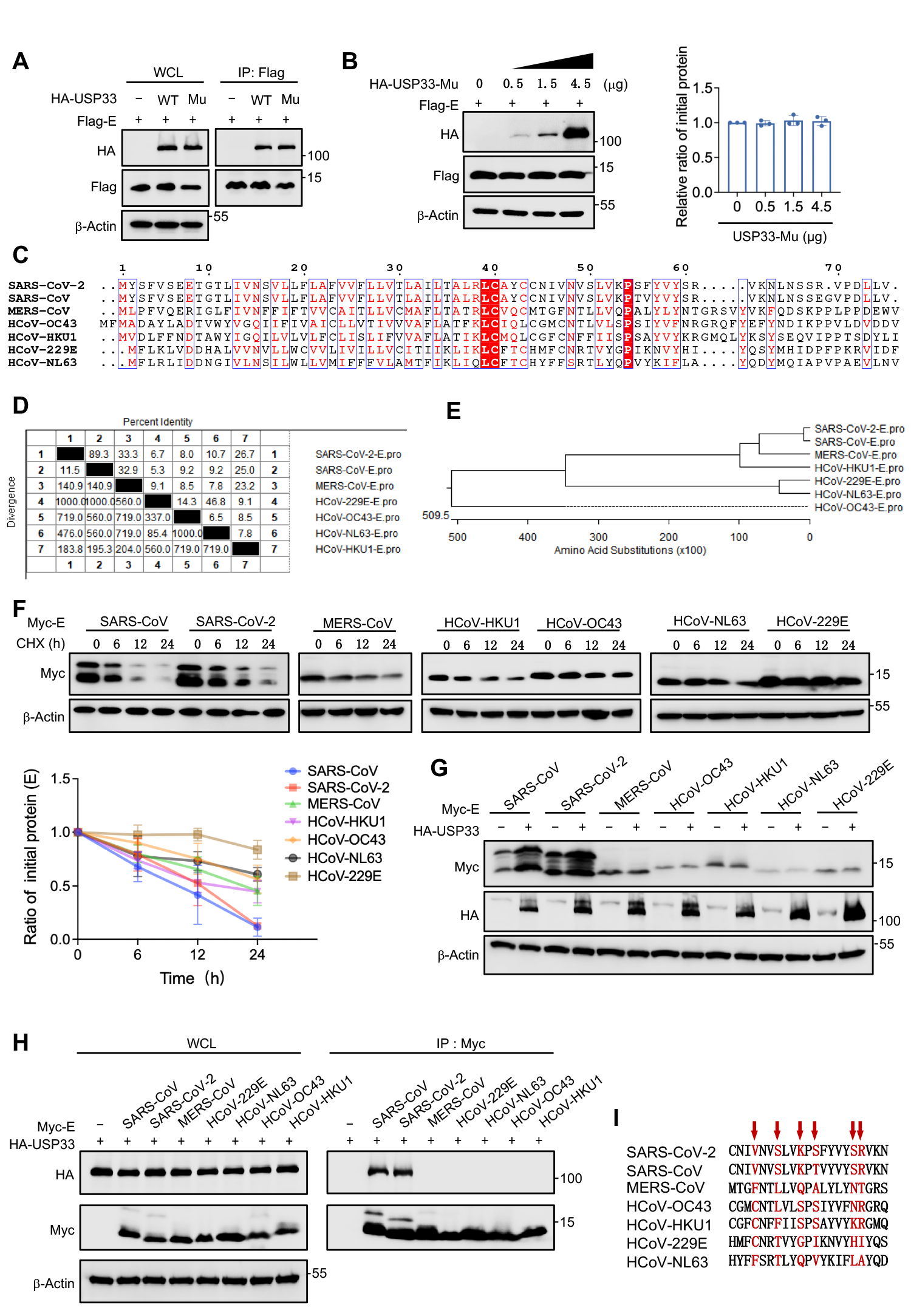
**

**Figure S8. Stability analyses of E proteins from different human coronaviruses (related to Figure 4).**

**(A).** The plasmids containing HA-USP33 and Flag-E were transfected into HEK293T cells as indicated. The WCLs were immunoprecipitated by anti-Flag beads, followed by immunoblotting.

**(B).** HEK293T cells expressing Flag-E were transfected with increasing amount of plasmids containing HA-USP33-Mu. The cells were lysed to detect the protein level of E by immunoblotting (left) and quantification relative to β-Actin was shown (right).

**(C).** Amino acid sequence alignments of seven human coronavirus E proteins.

**(D).** Amino acid sequence similarity of E protein in seven human coronaviruses.

**(E).** Homology of the E proteins from seven human coronaviruses evaluated using ClustalW.

**(F).** HEK293T cells transfected with plasmids expressing Myc-tagged E protein of seven human coronaviruses were treated with CHX for indicated time, and collected for immunoblotting to detect the protein levels of E (up). Quantification relative to β-Actin was shown as mean ± SD (n=3 independent experiments) (down).

**(G).** HEK293T cells were transfected with plasmids containing Myc-E and HA-USP33 as indicated. The protein levels were analyzed by immunoblotting.

**(H).** HEK293T cells transfected with plasmids containing Myc-E and HA-USP33 were lysed and immunoprecipitated with anti-Myc beads. The WCLs and precipitated proteins were analyzed by immunoblotting.

**(I).** Alignments of the interaction sites of E protein from seven human coronaviruses.

**Figure S9**

**
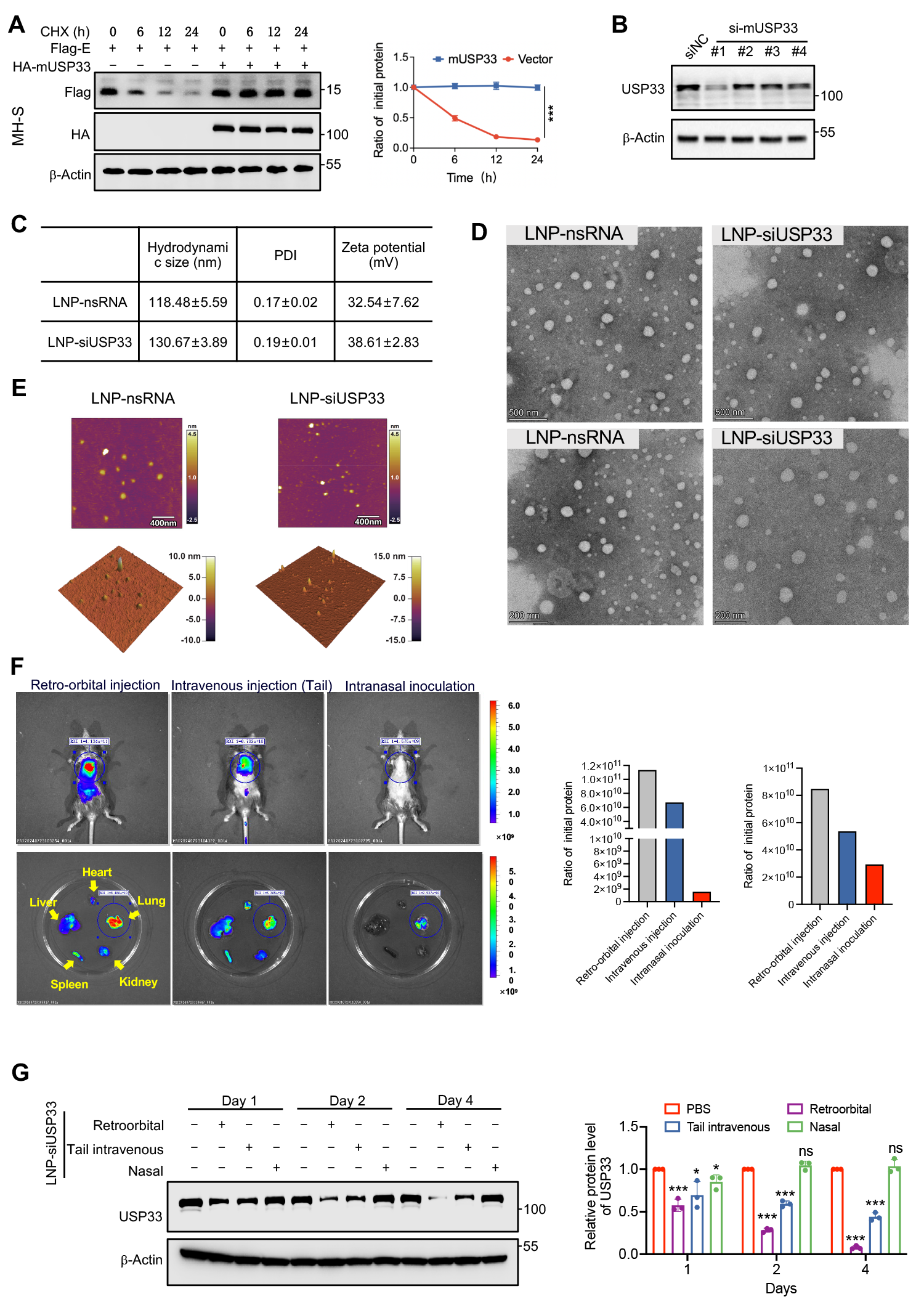
**

**Figure S9. LNP-encapsulated siUSP33 can be targeted for delivery to the lungs (related to Figure 5).**

**(A).** MH-S cells expressing Flag-E were transfected with empty vector or HA-mUSP33, treated with CHX and collected at indicated time for immunoblotting to analyze the protein level of E. The two-way ANOVA test was performed for comparisons of multiple groups. ****P*<0.001.

**(B).** Four siRNAs targeting mUSP33 and control siRNA were transfected into mouse alveolar macrophage MH-S, respectively. 48 h later the cells were collected to detect the protein level of USP33 by immunoblotting.

**(C).** The relevant parameters of the LNP-nsRNA and LNP-siUSP33, including Hydrodynamic size (nm), PDI and Zeta potential (mV).

**(D).** Representative transmission electron microscopic images of LNP-nsRNA and LNP-siUSP33. Scale bars, 500 nm (up) and 200 nm (down).

**(E).** The 3D reconstructed version of the image constructed by atomic force microscopy.

**(F).** The mice were administrated LNPs by retro-orbital injection, intravenous injection (Tail), and intranasal inoculation. The whole-body bioluminescence imaging and *ex vivo* fluorescence were shown (left) and quantified specially of the lung (right).

**(G).** The lungs of the mice with different treatment were collected on day 1, 2 and 4, followed by immunoblotting to detect the protein levels of USP33 (left). Quantification relative to β-Actin was shown as mean ± SD (n=3 independent experiments) (right). Student’s *t* *t*est (unpaired, two-tailed), **P*<0.05, ****P*<0.001, ns, not significant.

**Figure S10**

**
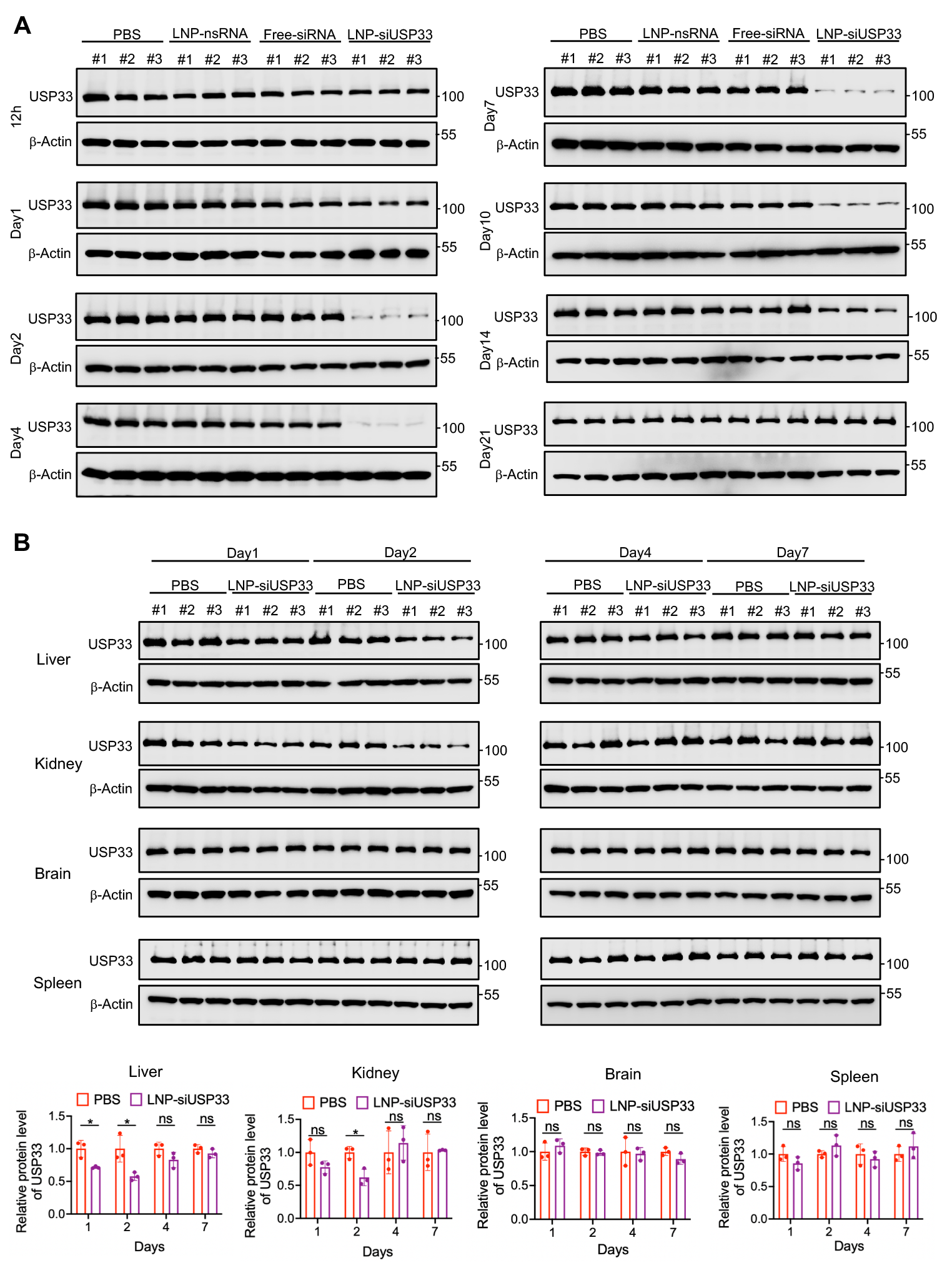
**

**Figure S10. Effect of targeted knockdown of LNP-siUSP33 in different mouse tissues (related to Figure 5).**

**(A).** The mouse lung tissues were collected at indicated time after injection, and lysed for immunoblotting to detect the knockdown efficiency of USP33.

**(B).** The livers, kidneys, brains and spleens were collected at indicated time after injection, and all the tissues were lysed for immunoblotting to detect the protein levels of USP33 (up). Quantification was shown as mean ± SD (n=3 independent experiments) (down). Student’s *t* *t*est (unpaired, two-tailed), **P*<0.05; ns, not significant.

**Figure S11**

**
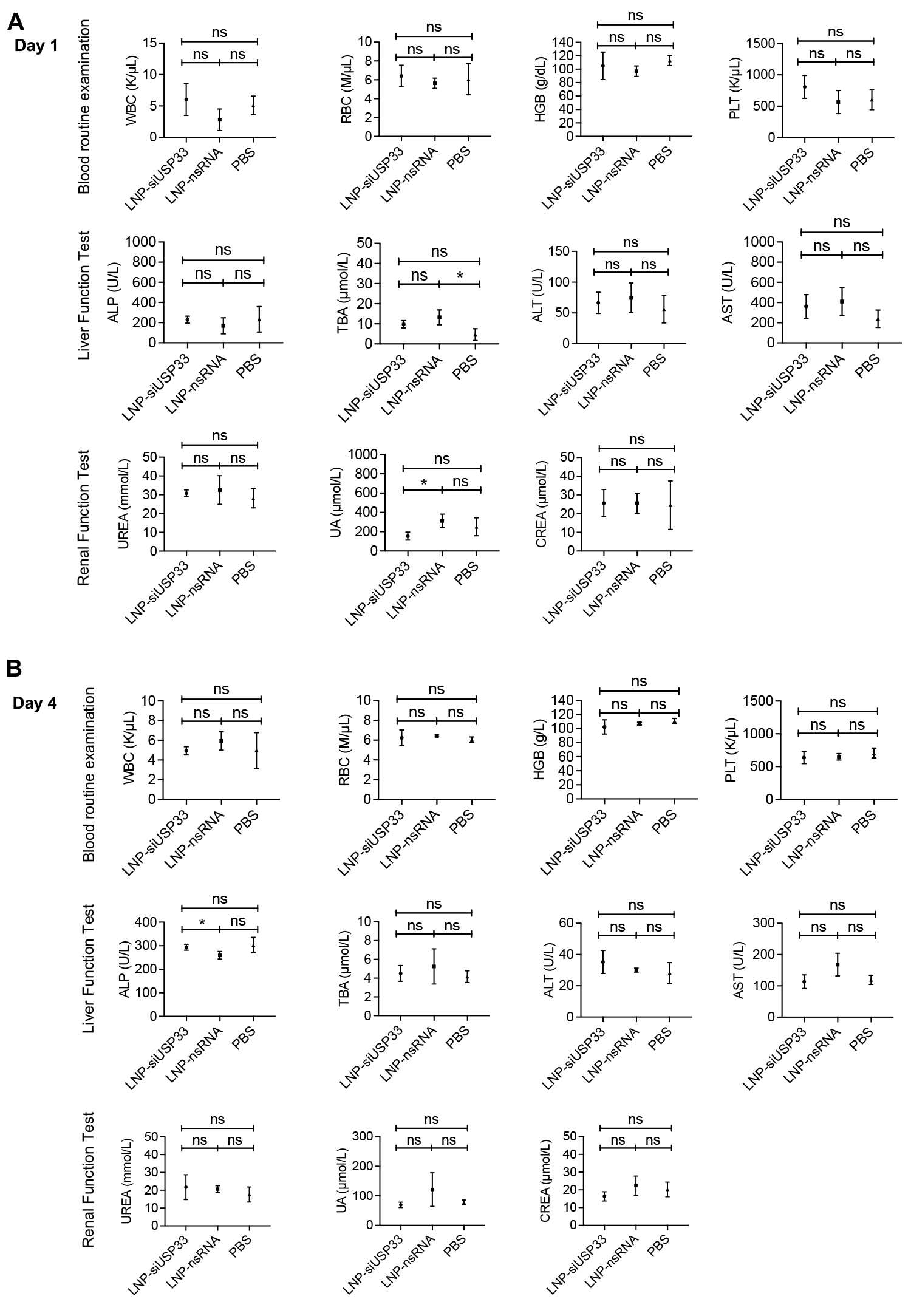
**

**Figure S11. Regular physiological indexes of mice in different treatment groups (related to Figure 5).**

**(A-B).** The peripheral blood of the mice with different treatment were collected on day 1 (A) and day 4 (B) to analyze the regular physiological indexes, including Blood routine examination (WBC, RBC, HGB, and PLT), Liver Function Test (ALP, TBA, ALT, and AST), and Kidney Function Test (UREA, UA, and CREA).

**Figure S12**

**
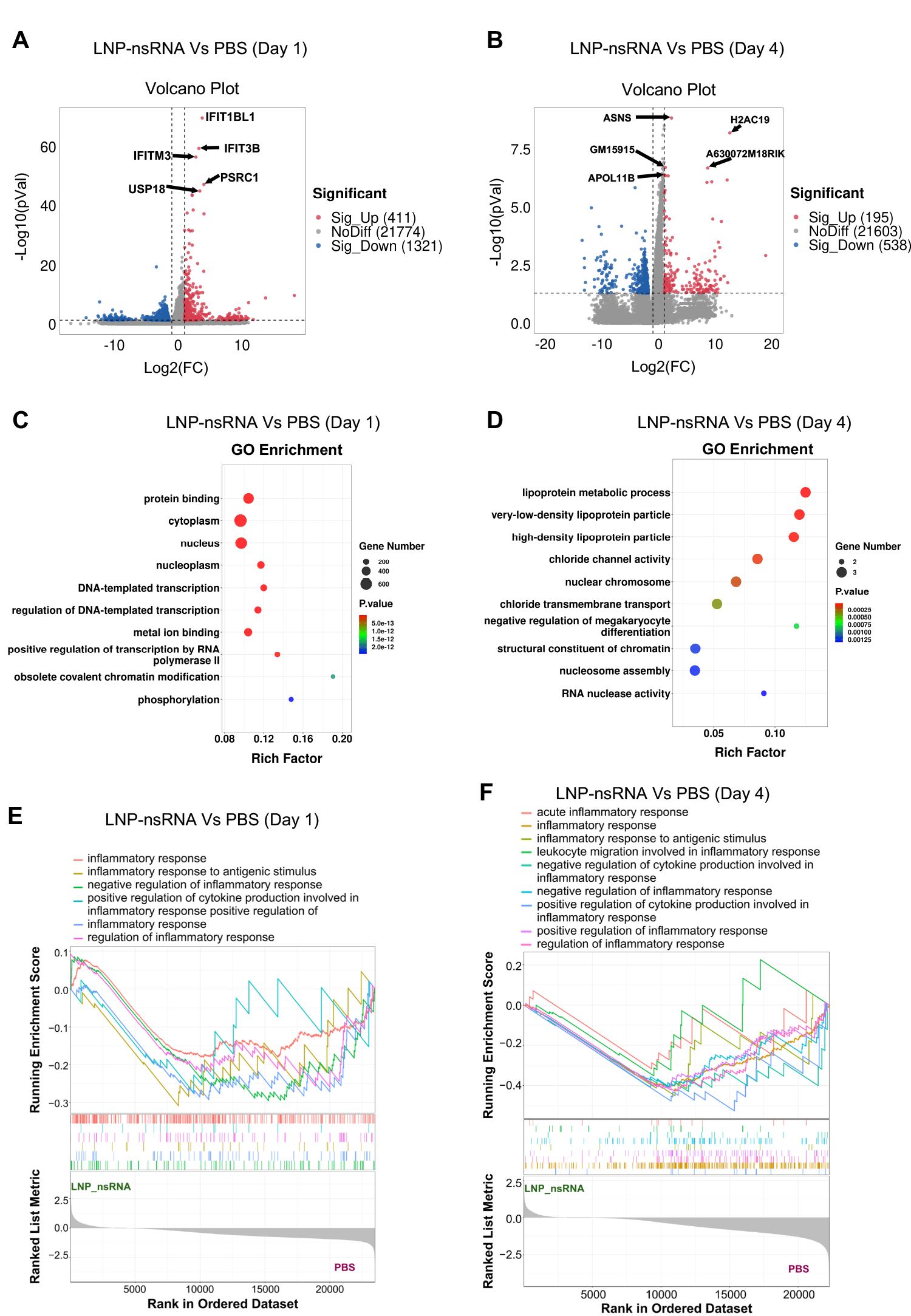
**

**Figure S12. The influence of LNP delivery on the peripheral blood transcriptome in mice (related to Figure 5).**

The peripheral blood of the mice in LNP-nsRNA and PBS treatment groups were collected for transcriptomics analysis. Volcano plot of changed genes on day 1 (A) and day 4 (B), GO enrichment of changed genes on day 1 (C) and day 4 (D), and Gene Set Enrichment Analysis of inflammation related genes on day 1 (E) and day 4 (F) were shown.

**Figure S13**

**
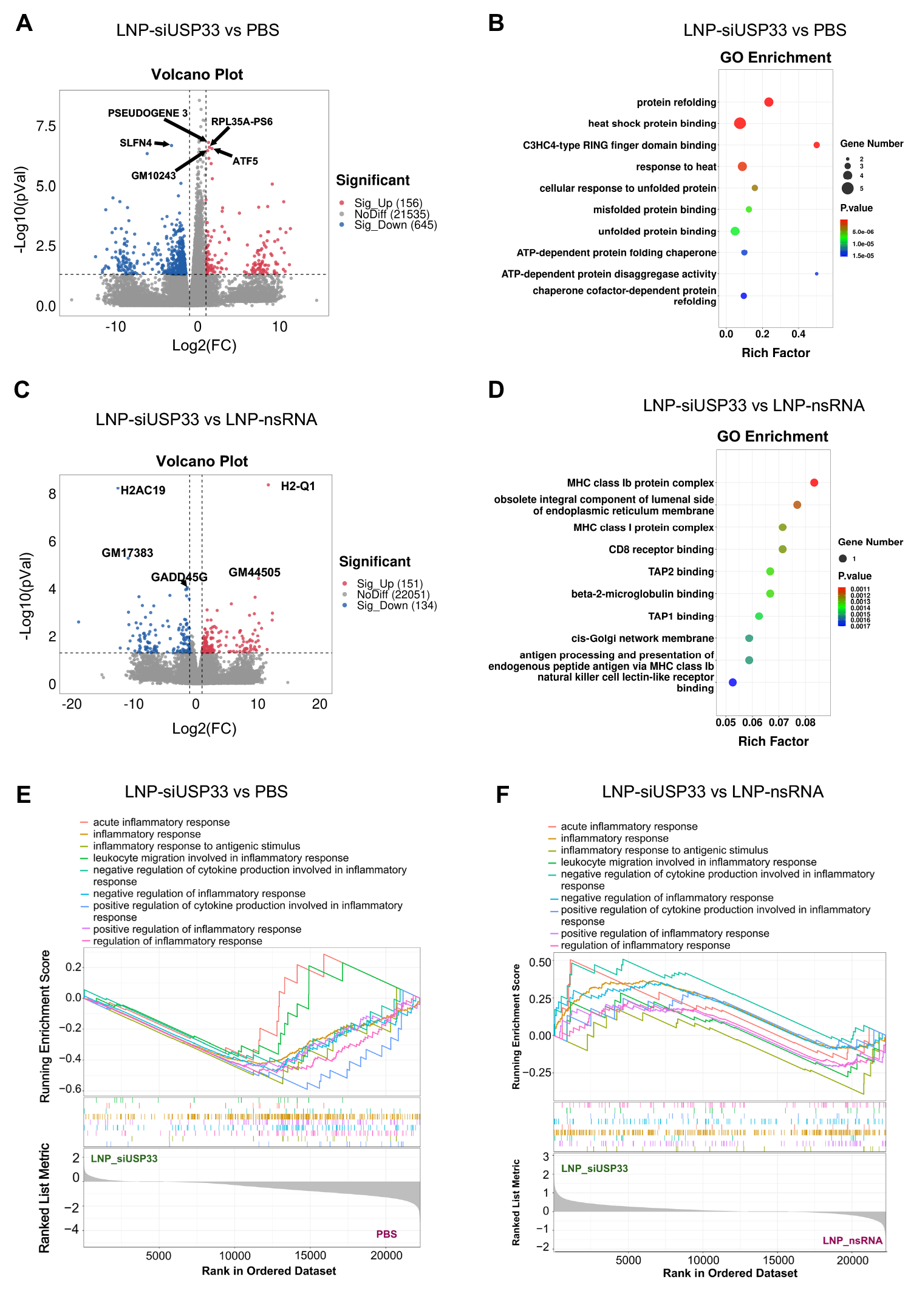
**

**Figure S13. The impact of USP33 knockdown on the peripheral blood transcriptome in mice (related to Figure 5).**

The peripheral blood of the mice in LNP-siUSp33, LNP-nsRNA and PBS treatment groups were collected on day 4 for transcriptomics analysis. Volcano plot of changed genes in LNP-siUSP33 group compared to PBS group (A), GO enrichment of changed genes in LNP-siUSP33 group compared to PBS group (B), Volcano plot of changed genes in LNP-siUSP33 group compared to LNP-nsRNA group (C), GO enrichment of changed genes in LNP-siUSP33 group compared to LNP-nsRNA group (D), Gene Set Enrichment Analysis of inflammation related genes in LNP-siUSP33 group compared to PBS group (E), and in LNP-siUSP33 group compared to LNP-nsRNA group (F) were shown.

**Figure S14**

**
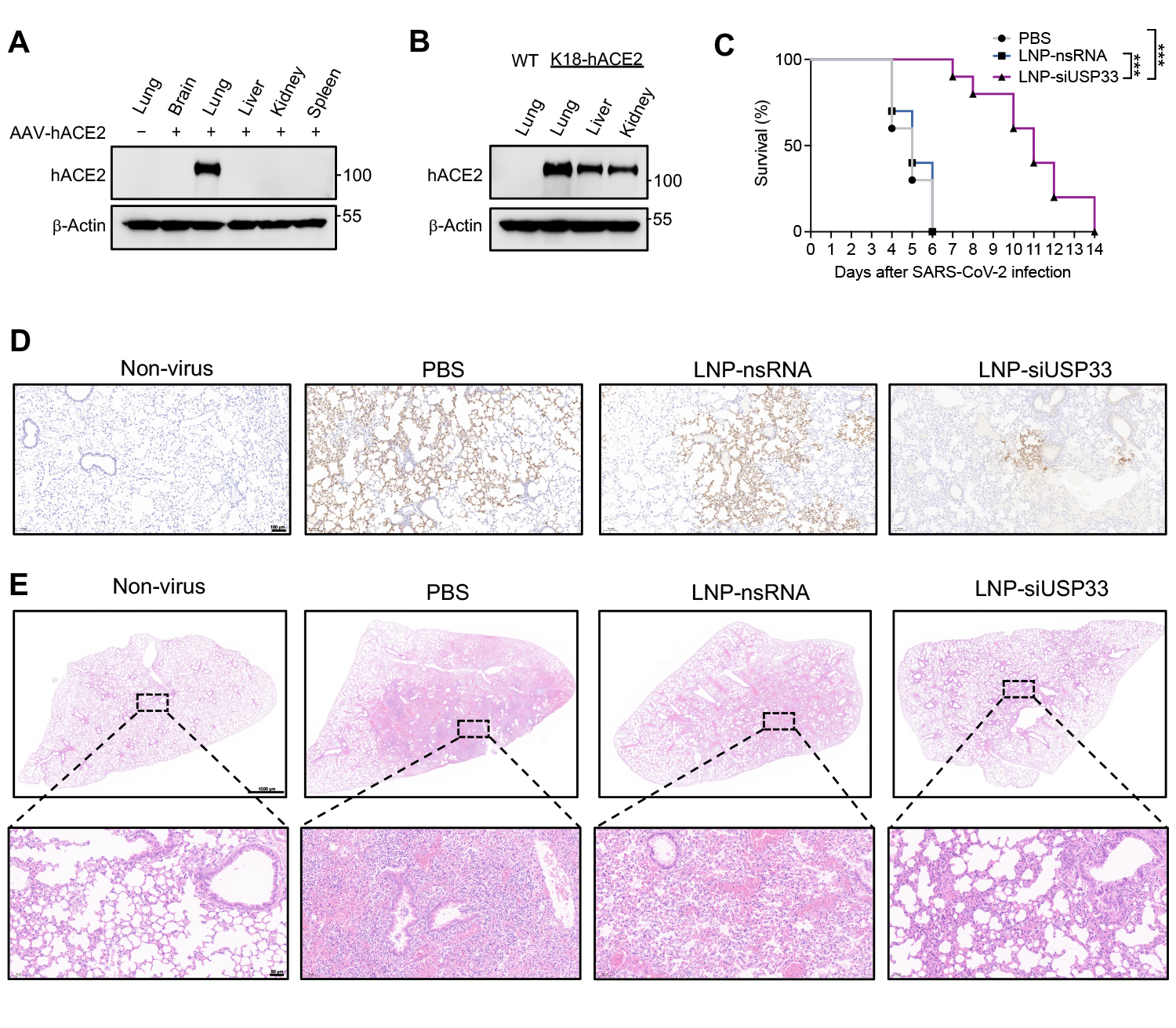
**

**Figure S14. LNP-siUSP33 reduces viral load and lung pathology in K8-hACE2 transgenic mice (related to Figure 6).**

**(A).** The indicated tissues of AAV-hACE2 transduced or not mice were collected， followed by immunoblotting to detect the hACE2 expression.

**(B).** The indicated tissues of WT and K18-hACE2 transgenic mice were collected， followed by immunoblotting to detect the hACE2 expression.

**(C).** Survival of K18-hACE2 transgenic mice after intranasal SARS-CoV-2 infection with different treatment (n = 10). The statistical significance of the survival curve was estimated according to the method of Kaplan and Meier, and the curve was compared with the generalized Wilcoxon test. ****P*<0.001

**(D).** Immunohistochemistry analysis with anti-SARS-CoV-2 N antibody was performed to assess the relative amount of SARS-CoV-2 in the lungs. Scale bar, 100 μm.

**(E).** H&E staining was performed to observe the intensity of the inflammatory infiltrate. Scale bars, 1000 μm (up) and 50 μm (down).

**Table S1. The Deubiquitinases library**

| Gene Name | NCBI Ref Seq | Gene Name | NCBI Ref Seq | Gene Name | NCBI Ref Seq | Gene Name | NCBI Ref Seq |
| --- | --- | --- | --- | --- | --- | --- | --- |
| USP1 | NM_001017415.1 | USP26 | NM_031907.3 | USP51 | NM_201286.3 | OTUB2 | NM_023112.3 |
| USP2 | NM_004205.4 | USP27X | NM_001145073.2 | USP53 | NM_019050.2 | OTUD1 | NM_001145373.2 |
| USP3 | NM_001256702.1 | USP28 | NM_001346258.1 | USP54 | NM_001320437.1 | OTUD3 | NM_015207.1 |
| USP4 | NM_001251877.1 | USP29 | NM_020903.2 | USPL1 | NM_005800.4 | OTUD4 | NM_001102653.1 |
| USP5 | NM_001098536.1 | USP30 | NM_032663.4 | PAN2 | NM_001127460.2 | OTUD5 | NM_001136157.1 |
| USP6 | NM_001304284.1 | USP31 | NM_020718.3 | CYLD | NM_001042355.1 | OTUD6A | NM_207320.2 |
| USP7 | NM_003470.2 | USP32 | NM_032582.3 | AMSH | NM_006463.4 | OTUD6B | NM_016023.3 |
| USP8 | NM_001128610.2 | USP33 | NM_015017.4 | AMSHLP | NM_020799.3 | OTUD7A | NM_130901.2 |
| USP9X | NM_001039590.2 | USP34 | NM_014709.4 | MYSM1 | NM_001085487.2 | OTUD7B | NM_020205.3 |
| USP9Y | NM_004654.3 | USP35 | NM_020798.2 | MPND | NM_001159846.2 | YOD1 | NM_018566.3 |
| USP10 | NM_005153.2 | USP36 | NM_001321291.1 | BRCC3 | NM_001018055.2 | ALG13 | NM_001039210.4 |
| USP11 | NM_004651.3 | USP37 | NM_020935.2 | STAMBP | NM_006463.4 | JOSD2 | NM_001270639.1 |
| USP12 | NM_182488.3 | USP38 | NM_001290325.1 | COPS5 | NM_006837.2 | JOSD1 | NM_014876.5 |
| USP13 | NM_003940.2 | USP39 | NM_001256725.1 | COPS6 | NM_006833.4 | ATXN3L | NM_001135995.1 |
| USP14 | NM_001037334.1 | USP40 | NM_018218.2 | PSMD7 | NM_002811.4 | ATXN3 | NM_001127696.1 |
| USP15 | NM_001252078.1 | USP41 | AJ583822.1 | PSMD14 | NM_005805.5 | BAP1 | NM_004656.3 |
| USP16 | NM_001001992.1 | USP42 | NM_032172.2 | EIF3H | NM_003756.2 | UCHL5 | NM_001199261.1 |
| USP17 | NM_201402.2 | USP43 | NM_001267576.1 | EIF3F | NM_003754.2 | UCHL3 | NM_006002.4 |
| USP18 | NM_017414.3 | USP44 | NM_001042403.2 | PRPF8 | NM_006445.3 | UCHL1 | NM_004181.4 |
| USP19 | NM_001199160.1 | USP45 | NM_001080481.1 | A20 | NM_024873.5 | MINDY1 | NM_001319998.1 |
| USP20 | NM_001008563.4 | USP46 | NM_001286767.1 | ZRANB1 | NM_017580.2 | MINDY2 | NM_001040450.2 |
| USP21 | NM_001014443.2 | USP47 | NM_001282659.1 | VCPIP1 | NM_025054.4 | MINDY3 | NM_024948.3 |
| USP22 | NM_015276.1 | USP48 | NM_001032730.1 | OTULINL | NM_019018.2 | MINDY4 | NM_032222.2 |
| USP24 | NM_015306.2 | USP49 | NM_001286554.1 | OTULIN | NM_138348.5 | MINDY4B | NM_001351281.2 |
| USP25 | NM_001283041.1 | USP50 | NM_203494.4 | OTUB1 | NM_017670.2 | ZUP1 | NM_145062.2 |

**Table S2. The shRNA and sgRNA sequences of this study**

| **Target gene** | **Sequence** |
| --- | --- |
| Human shUSP33#1 | 5’-GTGGAATTTGTCAGCAGATAT-3’ |
| Human shUSP33#2 | 5’-CCTCCGGTTGTTCATGTTGAT-3’ |
| Human shUSP33#3 | 5’-CTGGATATAGAAGCGGATGAA-3’ |
| Human shUSP33#4 | 5’-GCAACAGTGATAGAGCAGAAA-3’ |
| Human sgUSP33#1 | 5’-ACCATACTCGAAGAGTGGTA-3’ |
| Human sgUSP33#2 | 5’-ACCAAATCTTTGGGCATGTC-3’ |
| Human sgUSP33#3 | 5’-TGTCAGGATTGTAAAGTCCA-3’ |
| Human sgUSP33#4 | 5’-TGTCCACATTTGGATTCAGT-3’ |
| Human sgRNF5 | 5’-GCACCTGTACCCCGGCGGAA-3’ |

**Table S3. The primers for detecting mRNA level of genes in this study**

| **Target gene** | **Primer sequence (5’-3’)** |
| --- | --- |
| *Human* *CXCL10* | Forward: CACCATGAATCAAACTGCGA  Reverse: GCTGATGCAGGTACAGCGT |
| *Human IL6* | Forward: AGTTGCCTTCTTGGGACTGA  Reverse: TCCACGATTTCCCAGAGAAC |
| *Human* *IL8* | Forward: GGCACAAACTTTCAGAGACAG  Reverse: ACACAGAGCTGCAGAAATCAGG |
| *Human GAPDH* | Forward: AACTTTGGCATTGTGGAAGG  Reverse: ACACATTGGGGGTAGGAACA |
| *SARS-CoV-2 N* | Forward: GGCAGTAACCAGAATGGAGAACG  Reverse: ATGATGCCGTCTTTGTTAGCAC |
| *Mouse Tnfa* | Forward: TGGAACTGGCAGAAGAGGCACT  Reverse: GTAGACAGAAGAGCGTGGTGGC |
| *Mouse Il6* | Forward: CACTTCACAAGTCGGAGGC  Reverse: TTTGTATCTCTGGAAGTTTCAG |
| *Mouse Il1b* | Forward: TCGCAGCAGCACATCAACAAGA  Reverse: CCTGGAAGGTCCACGGGAAAGA |
| *Mouse Cxcl10* | Forward: GAACCCAAGTGCTGCCGTCATT  Reverse: CAGGATAGGCTCGCAGGGATGA |
| *Mouse Cxcl13* | Forward: TTGTGATCTGGACCAAGATGAA  Reverse: GACTTTTGCTTTGGACATGTCT |
| *Mouse Gapdh* | Forward: AGGTCGGTGTGAACGGATTTG  Reverse: GGGGTCGTTGATGGCAACA |
